# Characterizing the Organohalogen Iceberg: Extractable, Multihalogen Mass Balance Determination in Municipal Wastewater Treatment Plant Sludge

**DOI:** 10.1021/acs.est.3c01212

**Published:** 2023-06-12

**Authors:** Kyra M. Spaan, Bo Yuan, Merle M. Plassmann, Jonathan P. Benskin, Cynthia A. de Wit

**Affiliations:** † Department of Environmental Science, Stockholm University, Svante Arrhenius väg 8, SE-106 91 Stockholm, Sweden; ‡ Department of Chemistry, Norwegian University of Science and Technology, Høgskoleringen 5, N-7491 Trondheim, Norway

**Keywords:** EOX, CIC, PFAS, chlorinated paraffins, brominated flame retardants, sewage sludge

## Abstract

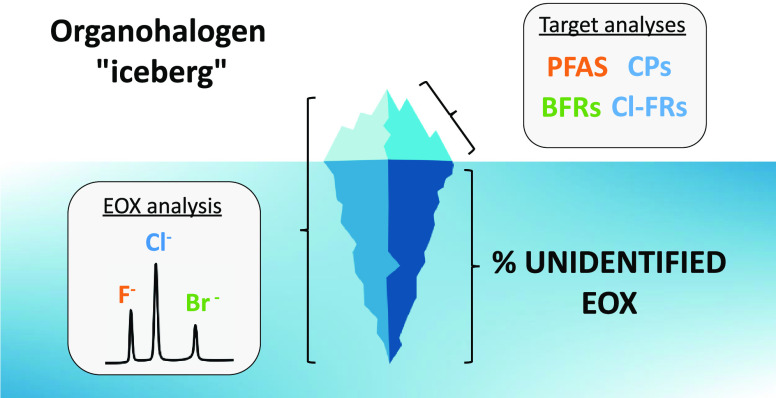

The large number and diversity of organohalogen compounds (OHCs) occurring in the environment poses a grand challenge to analytical chemists. Since no single targeted method can identify and quantify all OHCs, the size of the OHC “iceberg” may be underestimated. We sought to address this problem in municipal wastewater treatment plant (WWTP) sludge by quantifying the unidentified fraction of the OHC iceberg using targeted analyses of major OHCs together with measurements of total and extractable (organo)halogen (TX and EOX, respectively; where X = F, Cl, or Br). In addition to extensive method validation via spike/recovery and combustion efficiency experiments, TX and/or EOX were determined in reference materials (BCR-461 and NIST SRMs 2585 and 2781) for the first time. Application of the method to WWTP sludge revealed that chlorinated paraffins (CPs) accounted for most (∼92%) of the EOCl, while brominated flame retardants and per- and polyfluoroalkyl substances (PFAS) accounted for only 54% of the EOBr and 2% of the EOF, respectively. Moreover, unidentified EOF in nonpolar CP extracts points to the existence of organofluorine(s) with physical–chemical properties unlike those of target PFAS. This study represents the first multihalogen mass balance in WWTP sludge and offers a novel approach to prioritization of sample extracts for follow-up investigation.

## Abstract 2

A multihalogen mass balance experiment in WWTP sludge revealed high levels of unidentified organofluorine and organobromine. Organochlorine was characterized mainly by chlorinated paraffins.

## Introduction

Of the millions of chemicals used in society, organohalogen compounds (OHCs) are a class of particular concern. Approximately 30,000 individual OHCs are registered for production and use on the global market.^1^ The strength of the carbon–halogen bond imparts considerable stability and contributes to the environmental persistence, bioaccumulation, and toxicity of OHCs.^2^*In silico* screening of chemical inventories from Europe and North America revealed 3421 chemicals with both persistent and bioaccumulative properties, of which ∼52% were halogenated.^3^ Well-known classes of highly halogenated pollutants include per- and polyfluoroalkyl substances (PFAS), chlorinated paraffins (CPs), and halogenated flame retardants (HFRs) such as polybrominated diphenyl ethers (PBDEs) and organophosphate esters (OPEs). However, only a small fraction of OHCs are regulated by the global UN Stockholm Convention on Persistent Organic Pollutants; many others are considered trade secrets and are either undisclosed, ambiguously described, or infrequently monitored.^1^

Persistent OHCs used in society and industry eventually end up in wastewater and urban runoff, entering wastewater treatment plants (WWTPs). WWTP samples are therefore useful for monitoring stable chemicals used in society. Additionally, since sewage sludge may be released back into the environment via agriculture, recultivation/land reclamation, and landfilling (combined totaling ∼70% of all sludge in Europe^4^), it is of great importance to ensure that harmful substances are identified and prevented from entering the wastewater system so that sludge can be reused in a sustainable manner. Capturing the large number and diversity of OHCs in a single targeted method is currently not possible, leading to concerns that OHC contamination may be underestimated. For this reason, there is growing interest in so-called “organohalogen mass balance” experiments, which seek to quantify the fraction of unidentified organohalogen in samples. This is assessed through paired analysis of target OHCs and extractable organohalogen (EOX; where X = F, Cl, or Br).

The earliest organohalogen mass balance experiments date back to the mid 1990s and early 2000s. Instrumental neutron activation analysis (INAA) was used to determine EOCl, EOBr, and EOI across a wide range of environmental samples, including biota,^5−10^ incinerator ashes,^11^ air,^12^ and pine needles.^13^ These studies mostly targeted legacy pollutants, including polychlorinated biphenyls (PCBs), dichlorodiphenyl trichloroethanes (DDTs), hexachlorocyclohexanes (HCHs), polychlorinated/brominated dibenzo-p-dioxins (PCDDs/PBDDs), polychlorinated/brominated dibenzofurans (PCDFs/PBDFs), and PBDEs. More recently, PCBs, pesticides, and PBDEs were determined together with EOCl and EOBr in high trophic level mammals.^14^ In 2007, the first fluorine mass balance experiments were performed, pairing target PFAS analysis with combustion ion chromatography (CIC)-based EOF determination in water and human blood.^15,16^ Later, other matrices were analyzed, including biological samples,^17−22^ consumer products,^23,24^ and sewage sludge.^25−27^ Collectively, these studies point to significant gaps in the EOX mass balance across a diverse range of sample types. However, despite nearly three decades of research activity, there remains fairly little data on the contribution of contemporary OHCs (e.g., CPs and emerging HFRs) to the total OHC burden, and even fewer data on TX and EOX in reference materials, which are necessary for widespread adoption and standardization of halogen mass balance methods.

In the current study, we aimed to combine measurements of EOF, EOCl, and EOBr, with analysis of emerging OHCs to identify and compare the known and unknown fractions of the organohalogen “iceberg” in WWTP sludge. This approach enables prioritization of extracts/extraction procedures which generate the largest fractions of unidentified halogen for further investigation. To achieve this objective, we developed and validated a multihalogen CIC method for determination of EOX and total X (TX; where X= F, Cl, or Br). This method, together with quantitative target analyses of emerging fluorinated, chlorinated, and brominated organic contaminants (e.g., PFAS, CPs, and HFRs), was applied to form the first multihalogen mass balance data set in both municipal WWTP sludge and standard reference materials.

## Materials and Methods

### Sample Collection and Preparation

Digested (both aerobic and anaerobic) and dewatered sewage sludge was collected on November 14, 2019, at Henriksdal WWTP, situated in Stockholm, Sweden. The major treatment steps are illustrated in Figure S1 and include mechanical (coarse screens, grit chamber, primary sedimentation, sand filter), chemical (precipitation with iron), and biological (aerobic bioreactor, anaerobic digestion) treatments. The collected sludge is the final product after treatment. The Henriksdal WWTP has two inlets consisting mainly of domestic and commercial wastewater plus urban runoff, serving ∼870,000 people. The plant has an average sewage treatment capacity of 273,000 m^3^/day,^28^ and the average residence time of the sludge is 19 days. After sampling, the sludge samples were freeze-dried and homogenized. Water content, determined by noting the change in mass before and after freeze-drying, was 67%. Organic matter (64%) was determined by loss on ignition, in which the freeze-dried sludge sample was burned overnight at 105 °C followed by 2 h at 550 °C. Thereafter, organic carbon (39%) was estimated using a conversion factor of 0.6.^29^ The samples were stored in the freezer at −18 °C prior to analysis.

### Overview and Justification of Analytical Approach

While we initially considered a single holistic procedure for combined analysis of organofluorine, organochlorine, and organobromine substances, such a method is not currently available and would be extremely challenging to develop given the wide range of OHC physical–chemical properties and the vulnerability of OHC measurements to coextractable matrix interferences (which require treatment using specific cleanup procedures). Consequently, we focused our EOX mass balance on extracts produced from separate methods for measurement of three classes of OHCs: EOF/PFAS, EOCl/CPs, and EOCl+Br/HFRs. This approach compares EOX and target OHCs produced from the same (unique) extract and thus any gap between “known” and “total” EOX can reasonably be attributed to unknown substances with similar physical–chemical properties to the OHCs targeted by each method. We note that EOX is, by definition, an underestimate of “total organohalogen”, since some organohalogens are inevitably not extracted and/or are lost during cleanup. Targets were chosen due to their elevated concentrations in WWTP sludge,^27,30−34^ and by extension, expected contribution to the EOX mass balance. Moreover, targeted analytical methods for PFAS, CPs, and HFRs are well established in our laboratories. Other legacy halogenated classes such as PCBs and DDTs were not included, since their concentrations in previously analyzed sewage sludge from the same WWTP, sampled several times over the same year (2019) were consistently >45 times lower than medium chain length CPs (∑_7_PCBs at 21 ng/g dry weight [dw] compared to 972–3540 ng/g dw for MCCPs),^30^ and were not expected to contribute significantly to the organochlorine mass balance in this sludge sample.

**Figure 1:**
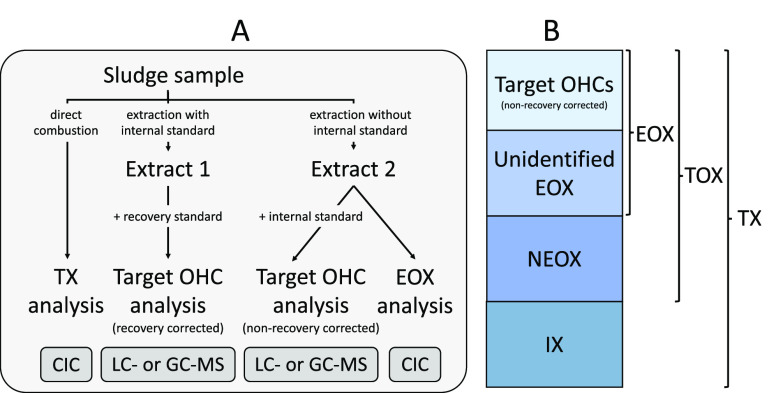
Experimental workflow for organohalogen mass balance analysis (A) and organohalogen mass balance concept (B). TX = total halogen, TOX = total organohalogen, EOX = extractable organohalogen, OHC= organohalogen compound, NEOX = nonextractable organohalogen (includes both nonextractable halogen as well as halogen removed during cleanup), IX = inorganic halogen.

Characterization of sludge samples involved four separate analyses for each OHC class (Figure 1A). First, TX concentrations, which represent the sum of all organic and inorganic halogen (IX), were determined by direct combustion (i.e., no sample preparation). Second, targeted OHC analysis was performed on replicate (*n* = 3) sludge samples which were spiked with internal standard (IS) and then extracted (performed on all but CPs; see details in section on CP Extraction Procedure) and cleaned up. Concentrations derived from these extracts (Extract 1 in Figure 1A) are corrected for procedural losses and are used to present target OHC profiles. In parallel, separate portions (*n* = 3) of the same sludge were extracted and cleaned up without IS, and the resulting extracts (Extract 2 in Figure 1A) were each split into two fractions: one fraction was spiked with IS postextraction and analyzed for target OHCs (nonrecovery corrected, representing the “known” fraction of EOX), while the second fraction was analyzed for EOX by CIC (no IS; Figure 1A). Target OHC and EOX concentrations in Extract 2 (both of which are nonrecovery corrected) are directly comparable and are therefore used to assess the halogen mass balance.

While the extraction and cleanup procedures employed here remove inorganic halogens (confirmed via spike/recovery experiments; see below), they may also selectively exclude some nonextractable organohalogens (NEOX; including OHCs that are nonextractable and/or removed during cleanup; Figure 1B). For this reason, EOX concentrations are expected to underestimate total organohalogen concentrations. Moreover, halogens that are not extractable by one method, may be observed in another. For example, an extremely nonpolar organofluorine that is not extractable using our PFAS extraction method (methanol) might be observable in CP extracts. For this reason, in addition to pairing EOX measurements with their associated targeted OHCs (i.e., PFAS/EOF, CPs/EOCl, and HFRs/EOCl+EOBr; see Table S1 for a full list of substances and abbreviations, and Figure S2 for schematic workflow), we also performed EOX measurements on several extracts where a particular halogen was unexpected (e.g., EOF measurements on CP extracts).

### PFAS Extraction Procedure

The PFAS extraction procedure was based on previous studies and is described in detail in the SI.^26,35^ Briefly, ∼0.5 g freeze-dried sludge was fortified with ISs and then extracted with methanol, followed by EnviCarb cleanup (Extract 1; Figure 1A). The same procedure was used for EOF mass balance analysis (Extract 2; Figure 1A), but ISs were not added prior to extraction. Extract 2 was split: one portion was fortified with ISs for target PFAS analysis while the remainder was stored for EOF determination by CIC. Instrumental analysis for target PFAS was performed by ultraperformance liquid chromatography–tandem mass spectrometry (UPLC-MS/MS) and included 11 perfluoroalkyl carboxylic acids (PFCAs), 4 perfluoroalkyl sulfonic acids (PFSAs), and perfluorooctane sulfonamide (FOSA). Instrumental details are presented in our previous study,^21^ and retention times, precursor and selected ions, ISs, and limits of quantification (LOQs) are presented in Table S2. Accuracy and precision of targeted analysis was assessed via replicate (*n* = 3) spike/recovery experiments for nonrecovery corrected Extract 2, performed by adding 5 ng of individual PFAS into sludge and analyzing them together with real samples (Table S3). Additionally, replicate (*n* = 3) portions of standard reference material (SRM) 2781 (“domestic sludge”, NIST, Gaithersburg, MD), with reported values for several PFAS, were analyzed for method performance comparison (Table S4). Additional QC and method validation procedures are described below.

### CP Extraction Procedure

The CP extraction procedure was based on accelerated solvent extraction (ASE) using an ASE350 (Dionex, US) using a previously developed method (see SI for details).^36^ A separate targeted analysis (i.e., Extract 1 in Figure 1A; with ISs added prior to extraction) was not included because prior data have shown IS recoveries close to 100% (i.e., non-IS corrected values can be expected to be the same as IS-corrected values).^37^ Briefly, ∼0.5 g portions of freeze-dried sludge were loaded into ASE cells. Extraction was performed at 100 °C and 1500 psi using *n*-hexane:acetone (1:1) as the extraction solvent. The resulting extract was evaporated to near dryness with nitrogen and underwent three cleanup steps for lipid and elemental sulfur removal. The final extract was concentrated to ∼1 mL, after which a portion (150 μL) was spiked with 10 ng ^13^C-labeled C_10_Cl_6_ for target CP analysis; the remainder of the extract was analyzed for EOCl content by CIC. Targeted analysis of very short-, short-, medium-, and long-chained CPs (vSCCPs, SCCPs, MCCPs, LCCPs) was carried out by UPLC–atmospheric pressure chemical ionization–high resolution mass spectrometry (APCI-HRMS) using a QExactive HF Orbitrap (Thermo Fisher Scientific) in full scan (*m*/*z* 250–2000) mode with a resolution of 120,000 full width at half-maximum (fwhm). A total of 342 CP homologues (expressed as C_n_Cl_m_, n = 6–33 m = 1–16) were measured. Instrumental details are described elsewhere.^38^ Quantification was carried out based on a C_n_Cl_m_-profile reconstruction method by Bogdal et al.^39^ Sixteen commercial mixtures were used for quantification. The chlorine weight was calculated based on the semiquantitative determination of the degree of chlorination obtained by the instrument. The deviation from the manufacturer’s values ranged from 0.6% to 2.5% Cl. LOQs were calculated from the average blanks levels plus 10 times the standard deviations (Table S5). Accuracy and precision of CP determination were assessed via spike/recovery experiments using portion of diatomaceous earth (*n* = 3) fortified with 196, 1736, and 1068 ng of SCCPs (55.5% Cl), MCCPs (52.0% Cl), and LCCPs (49% Cl), respectively (Table S6). Replicates (*n* = 3) of NIST SRM 2585 “organic contaminants in house dust” (NIST, Gaithersburg, MD) were also analyzed and compared to previous studies (Table S7). Additional QC and method validation procedures are described below.

### HFR Extraction Procedure

For target HFR analysis, 2 g of freeze-dried sludge was weighed into a tube, fortified with isotope-labeled ISs, and then extracted using a previously published method (details in SI).^40^ Thereafter, fractionation of the extract (Extract 1, Figure 1A) was performed using a silica column based on a previously published method^41^ with slight modifications (see SI for details). The analytes were eluted in three fractions that were subsequently cleaned up individually (Figure S3), prior to targeted analysis and EOX. Fraction I contained PBDEs, decabromodiphenyl ethane (DBDPE), and some emerging brominated flame retardants (BFRs). Fraction II included 2-ethylhexyl 2,3,4,5-tetrabromobenzoate (EH-TBB), 1,2-bis(2,4,6-tribromophenoxy)ethane (BTBPE), and bis(2-ethylhexyl) tetrabromophthalate (BEH-TEBP). Fraction IIIa contained tetrabromobisphenol A (TBBPA), α- β-, and γ-1,2,5,6,9,10-hexabromocyclododecane (HBCDD), and Fraction IIIb contained chlorinated OPEs. Fractions I and II were analyzed on a gas chromatography mass spectrometer (GC-MS) equipped with an electron capture negative ionization (ECNI) source. Since highly brominated PBDEs are prone to thermal degradation,^42^ the octaBDEs, nonaBDEs, and decaBDEs were analyzed using a short column (15 m), while the remaining BDEs were analyzed on a 30 m column. Fraction IIIa was run on a LC-MS/ESI. Fraction IIIb analysis was carried out on a GC-MS coupled to an electron ionization (GC-MS/EI), as described previously.^41^ Bromine and chlorine mass balance analysis was performed using the aforementioned method, but extracts (Extract 2, Figure 1A) were only fortified with ISs immediately prior to instrumental analysis. Retention times and LOQs are available in the SI (Table S8). IS yields in blanks and sludge samples are presented in Table S9. For targets with IS recoveries <20% or >150% (i.e., TBBPA, HBCDDs, and TCEP), no quantification was performed. Accuracy and precision in the nonrecovery corrected Extract 2 were evaluated using replicate (*n* = 3) spike/recovery experiments performed at concentrations ∼5 times higher than the measured/expected concentration in sludge (Table S10). Additional QC and method validation procedures are described below.

### TX and EOX Analyses

TX and EOX measurements were performed using a CIC (Thermo-Mitsubishi), controlled by NSX-2100 software. Neat samples (∼5 mg) or extracts (100–300 μL) were placed on a ceramic boat (for extracts, the boats contained glass wool for better dispersion). The boats, including glass wool, were baked prior to use. The samples were combusted slowly in a combustion furnace (HF-201, Mitsubishi) at 1100 °C under a flow of oxygen (300 mL/min) and argon mixed with water vapor (200 mL/min) for approximately 5 min. Combustion gases were absorbed in Milli-Q water during the entire length of the combustion process using a gas absorber unit (GA-210, Mitsubishi). A 200 μL aliquot of the absorption solution was subsequently injected onto an ion chromatograph (Dionex Integrion HPIC, Thermo Fisher Scientific) equipped with an anion exchange column (2 mm × 50 mm guard column (Dionex IonPac AS19-4 μm) and 2 mm × 250 mm analytical column (Dionex IonPac AS19-4 μm)) operated at 30 °C. Chromatographic separation was achieved by running a gradient of aqueous hydroxide mobile phase ramping from 8 to 60 mM at a flow rate of 0.25 mL/min (Table S11), and halogens were detected using a conductivity detector. Retention times were approximately 7.5, 11.5, and 14.4 min for F^–^, Cl^–^, and Br^–^, respectively.

CIC data were processed using Chromeleon 7.2, and quantification was performed using a dilution of the inorganic standard in deionized water (Combined Seven Anion Standard I, #056933, Dionex). The 9-point calibration ranged from 0.05 to 20 ng/μL for F^–^, 0.075–30 ng/μL for Cl^–^, and 0.25–100 ng/μL for Br^–^ and showed good linearity (R^2^ > 0.99). The mean concentrations in the boat blanks were subtracted from the samples. The instrumental detection limit (DL), defined as the mean concentration plus three times the standard deviation in the boat blanks, was 0.11 ng/μL for F^–^ and 0.34 ng/μL for Cl^–^. Since Br^–^ was not detected in instrumental blanks, it was determined by the lowest detectable calibration point at 0.25 ng/μL.

The quantified target OHC levels were converted to halogen equivalents (i.e., mass of halogen per mass of sample [ng X/g]) according to eq 1, where C_OHC_ is the concentration of OHC (ng/g), n_X_ is the number of halogens in the molecule, A_X_ is the atomic mass of the halogen (g/mol), and MW_OHC_ is the molecular weight of the OHC (g/mol).
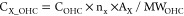
1

### Quality Control, Method Validation, and Reference Material Characterization

Halogen mass balance experiments require careful quality control and method validation in order to ensure comparability between target OHCs, EOX, and TX data. While reference materials are available for validating target OHC measurements across a wide range of matrices, very few of these materials exist for TX or EOX determination. In the present study, we performed a series of method validation experiments in order to ensure high quality halogen mass balance data. Moreover, we provide here some of the first TX and EOX data on commercially available standard reference materials (SRMs), in order to facilitate interlaboratory comparison and standardization of halogen mass balance experiments. An overview of both QC and method validation experiments, as well as reference material characterization is provided here:

- **Accuracy/precision:** Replicate spike/recovery experiments were carried out for most target OHCs (details in individual Materials and Methods section). In addition, NIST SRM 2585 (house dust), NIST SRM 2781 (domestic sludge), and BCR-461 (fluorine in clay) were analyzed for CPs, PFAS, and TF, respectively, and compared to reference values, including an interlaboratory comparison on EOF measurements.^26^ Finally, a comparison of target data generated with and without fortification of ISs prior to extraction was included for most targets (except CPs).
- **Removal of inorganic halogens:** In order to ensure efficient removal of inorganic halogens during the extraction procedures, QC samples were prepared using sludge fortified with NaF (50 ng, *n* = 3; F mass balance experiments) or NaCl (250 μg, *n* = 3; Cl mass balance experiments using the CP extraction method) and analyzed together with real samples. Additionally, a spike/recovery experiment was performed with blanks, nonspiked sludge, NaF spikes of 250 μg F^–^, and 23 mg F^–^ onto sludge. Also, a water extraction of the sludge was tested. The F^–^ concentration was measured with a fluoride ion selective electrode (ISE).
- **Halogen specificity (CIC analysis):** Combustion efficiencies for two OHCs per halogen class were tested (PFOA and PFOS; TCIPP and TDCPP; BDE183, and BDE209) at a range of concentrations, by quantifying combusted standard solutions using an inorganic halogen calibration curve. Recoveries were calculated by dividing the measured concentration by the expected concentrations ×100%.
- **Monitoring laboratory background contamination:** Replicate (*n* = 2 or 3) procedural blanks were prepared and analyzed together with samples in each extraction method in order to monitor lab background levels.
- **TX and EOX determination in SRMs:** The following SRMs were characterized for TX and EOX: Sludge NIST SRM 2781: TF, TCl, TBr and EOF; Dust NIST SRM 2585: TF, TCl, TBr, EOF, EOCl and EOBr; Clay BCR-461: TF, TCl and TBr.

## Results and Discussion

### Quality Control of Targeted Analyses

For targeted PFAS analysis, procedural blanks were negligible, and accuracy and precision of spiked samples were reasonable for most substances (61%–89%; RSD: 7%–33%) with the exception of PFTrDA and PFTeDA (249% and 324%, respectively; RSD: 19% and 14%; Table S3). Analysis of NIST sludge SRM 2781 resulted in target PFAS concentrations similar to NIST values (i.e., 36%–91%), despite fortifying with ISs after extraction (Table S4). For targeted analysis of CPs, procedural blank contamination was negligible relative to sludge (i.e., 3.4, 9.4, and 0.2 ng/g for SCCPs, MCCPs, and LCCPs, respectively), and therefore, blank subtraction was not performed. Spike/recovery experiments produced average CP percent recoveries of 89 ± 9%, 109 ± 10%, and 102 ± 3% for SCCPs, MCCPs, and LCCPs, respectively, demonstrating excellent method accuracy and precision (Table S6). Although there is no certified reference material available for CPs, CP concentrations in house dust SRM 2585 have been reported previously, and concentrations determined here (6.5 ± 0.2, 11.0 ± 0.4, and 19.6 ± 0.2 μg/g for SCCPs, MCCPs, and LCCPs, respectively) were similar to other studies (Table S7).^43−45^

For targeted analysis of chlorinated flame retardants, IS recoveries were acceptable (46%; RSD: 23%) for TDCPP, but low (<20%) for TCEP; consequently, only the former target was quantified. TCIPP blank levels (22.6 ng) were significant and were therefore subtracted from the sludge samples. Finally, for targeted analysis of BFRs, no contamination was detected in the procedural blanks. Spike/recovery results for BFRs generally ranged from 68% to 133% (RSD 2%–26%), indicating reasonable accuracy and precision in most cases (Table S10). Exceptions were BDE196, BDE206, TBP-AE, BTBPE, and BEH-TEBP, which showed lower recoveries (38%, 59%, 6%, 42%, 22%, respectively), and DBDPE, PBBz, PBT, TBP-DBPE, HBB, DBHCTD, and DDC-CO (syn), which showed higher recoveries (178%, 199%, 261%, 251%, 407%, 161%, respectively), likely due to matrix effects. Nevertheless, most of these over- and under-recovered targets are not likely to affect the EOBr mass balance, since they account for a small proportion of the EOBr.

Overall, these results point to reasonable method accuracy and precision across all target OHCs included in the present work, providing confidence in assessment of known EOX concentrations in sludge samples.

### Quality Control of Halogen Mass Balance

Samples spiked with inorganic halogens displayed percent recoveries of <4% for NaF (PFAS extraction) and <0.1% for NaCl (CPs extraction), with measured EOX concentrations that were not significantly different from unspiked samples. Fluoride concentrations were also measured in the final sludge extract using a fluoride ISE, and were not statistically different from the procedural blank (0.017 ± 0.006 vs 0.010 ± 0.002 μg F/g, respectively), nor did they make a substantial contribution to EOF (<5.6%; Table S12). Nevertheless, caution is warranted when using this extraction method procedure, since high-fluoride content samples could lead to elevated fluoride in the final extract (Table S12).

**Figure 2:**
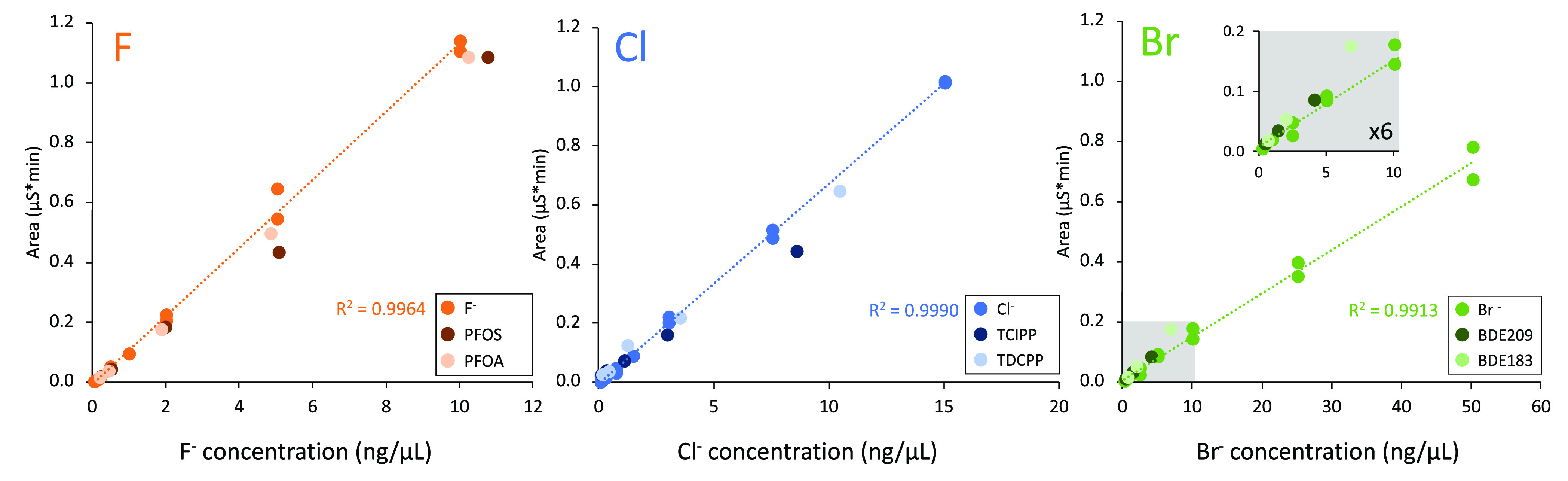
Plots showing consistent combustion efficiencies (i.e., halogen-specific response) at different concentrations for both inorganic and organic halogen standards.

Combustion efficiencies among organic and inorganic halogen standards, as well as in the presence of matrix (BCR-461 clay) revealed consistent halogen-specific responses (Figure 2; Table S13), with most combustion efficiencies ranging from 84 ± 16% (TClPP) to 110 ± 31% (BDE209), and good accuracy of 104 ± 4% found for BCR-461 (fluorine only). The exception was for BDE183 which displayed a slightly higher and more variable combustion efficiency (137 ± 40%) at low concentrations. These data are in reasonable agreement with a prior study investigating combustion efficiencies of 13 PFAS, which showed consistent combustion efficiency for most substances.^46^ Overall, combustion efficiencies for inorganic and organic F, Cl, and Br were sufficiently accurate and precise for carrying out OHC mass balance experiments, although there is a need for EOX interlaboratory studies and reference materials to further standardize these methods for widespread use.

### Characterization of Standard Reference Materials

Direct combustion of NIST domestic sludge SRM 2781 resulted in concentrations of 813, 2225, and 120 μg X/g dw for F^–^, Cl^–^ and Br^–^, respectively, and in NIST house dust SRM 2585, TX concentrations were 315 ± 8, 8820 ± 94, and 471 ± 111 μg X/g dw, F^–^, Cl^–^, and Br^–^, respectively (Table 1). In addition, TCl and TBr measurements in BCR-461 clay (which is certified for total fluorine) produced concentrations of 69 ± 9 μg Cl/g dw and nondetectable levels of Br. To the best of our knowledge, this is the first time NIST SRMs have been characterized for total halogens. Since TX measurements do not require sample handling, these data enable interlaboratory comparison and standardization of instrumental analysis methods.

**Table 1:**
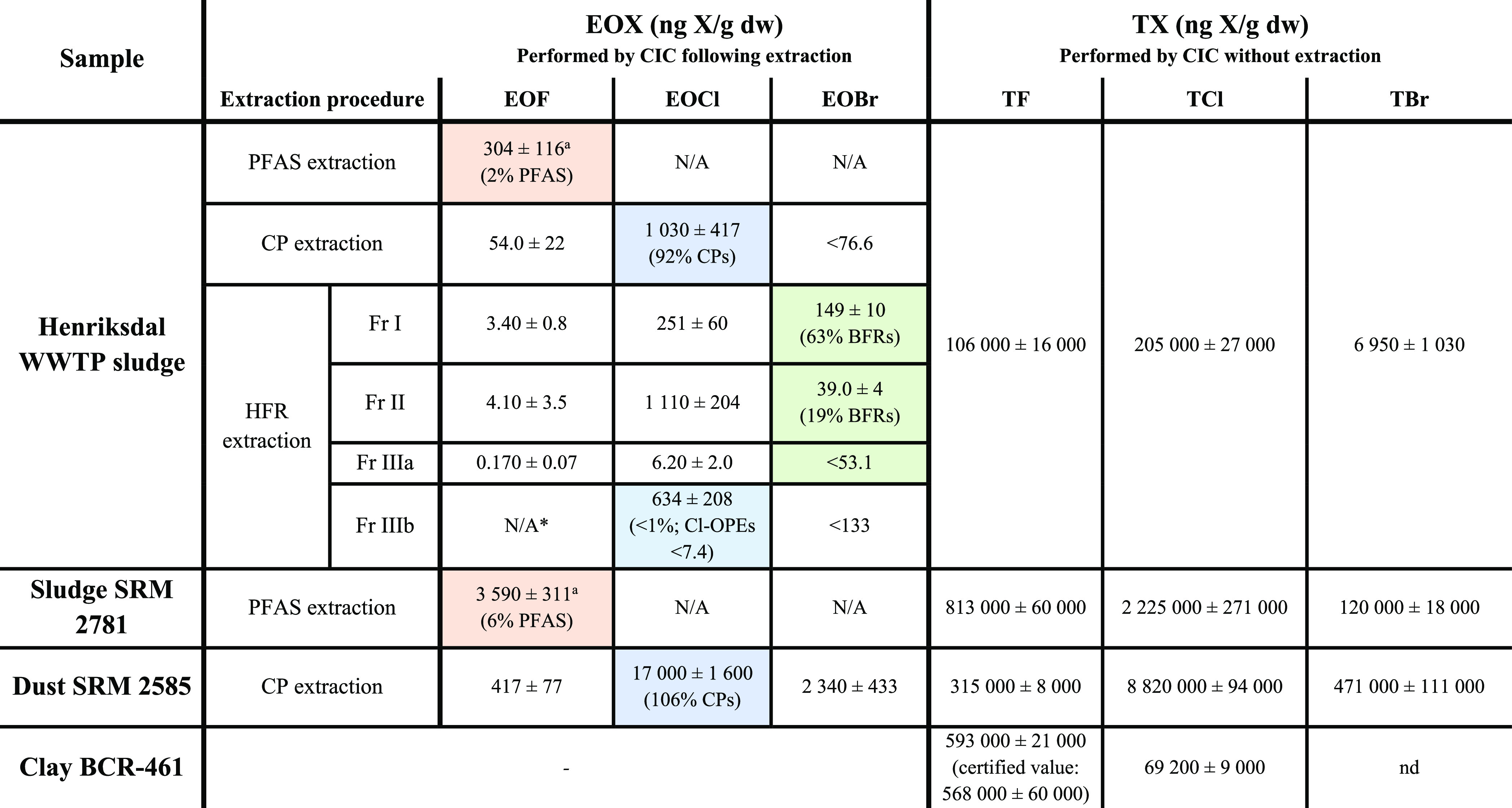
EOX and TX Results for WWTP Sludge, NIST SRMs, and BCR-461*

* Samples where organohalogen mass balance was performed are highlighted, and percentages indicate the fraction of EOX that was characterized. Individual target analyte concentrations are presented in Table S16. ^a^Extraction blank subtracted (−47 ng F/g). N/A = not analyzed. N/A* = not analyzed due to high instrumental blank levels.

In addition to TX determination, organohalogen mass balance measurements were carried out on NIST reference materials for the first time. SRM 2781 (sludge) was extracted with the PFAS extraction method and contained 3590 ± 311 ng F/g dw of EOF, of which ∼6% (i.e., 226 ± 10 ng F/g) was accounted for by the 16 PFAS included in the present work (Table 1; Table S4). SRM 2585 (dust) extracted using the CP extraction procedure contained 417 ± 77 ng F/g dw, 17,000 ± 1600 ng Cl/g dw, and 2340 ± 433 ng Br/g dw. Essentially all (∼106%; 17,900 ± 300 ng Cl/g) of the EOCl was accounted for by CPs (Table 1; Table S7). We note that there are a number of other chlorinated chemicals with certified or reference values present in this SRM (e.g., PCBs, DDE and DDT, chlordane, heptachlor, and nonachlor), which when summed produce a chlorine equivalent of ∼1.8 μg Cl/g dw (i.e., ∼11% of EOCl in the CP extract) and therefore would contribute considerably less than CPs. Furthermore, these compounds might not be extracted with the CP extraction method employed here. Finally, it is germane to note that EOX measurements determined using extraction methods other than those employed in the present work may generate different results. Nevertheless, these measurements represent a first step toward standardizing EOX measurements and will aid in interlaboratory comparison and external quality control.

### Halogen Mass Balance in Sewage Sludge

#### PFAS

**Figure 3:**
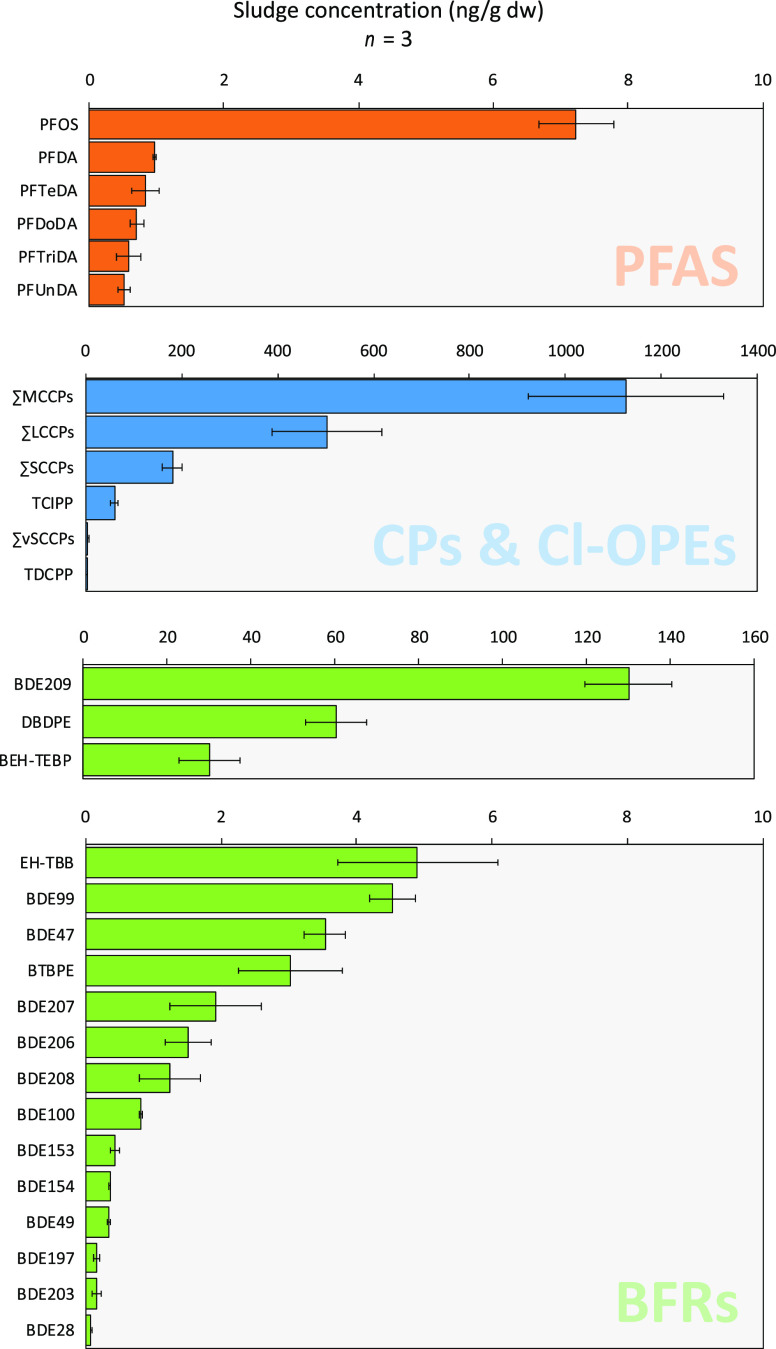
Target analyte concentrations in sludge (*n* = 3; recovery corrected) for fluorinated, chlorinated, and brominated compounds. Note the two concentration axes for BFRs. Error bars indicate standard deviation of replicate extractions. The full target list including abbreviations is shown in Table S1.

**Figure 4:**
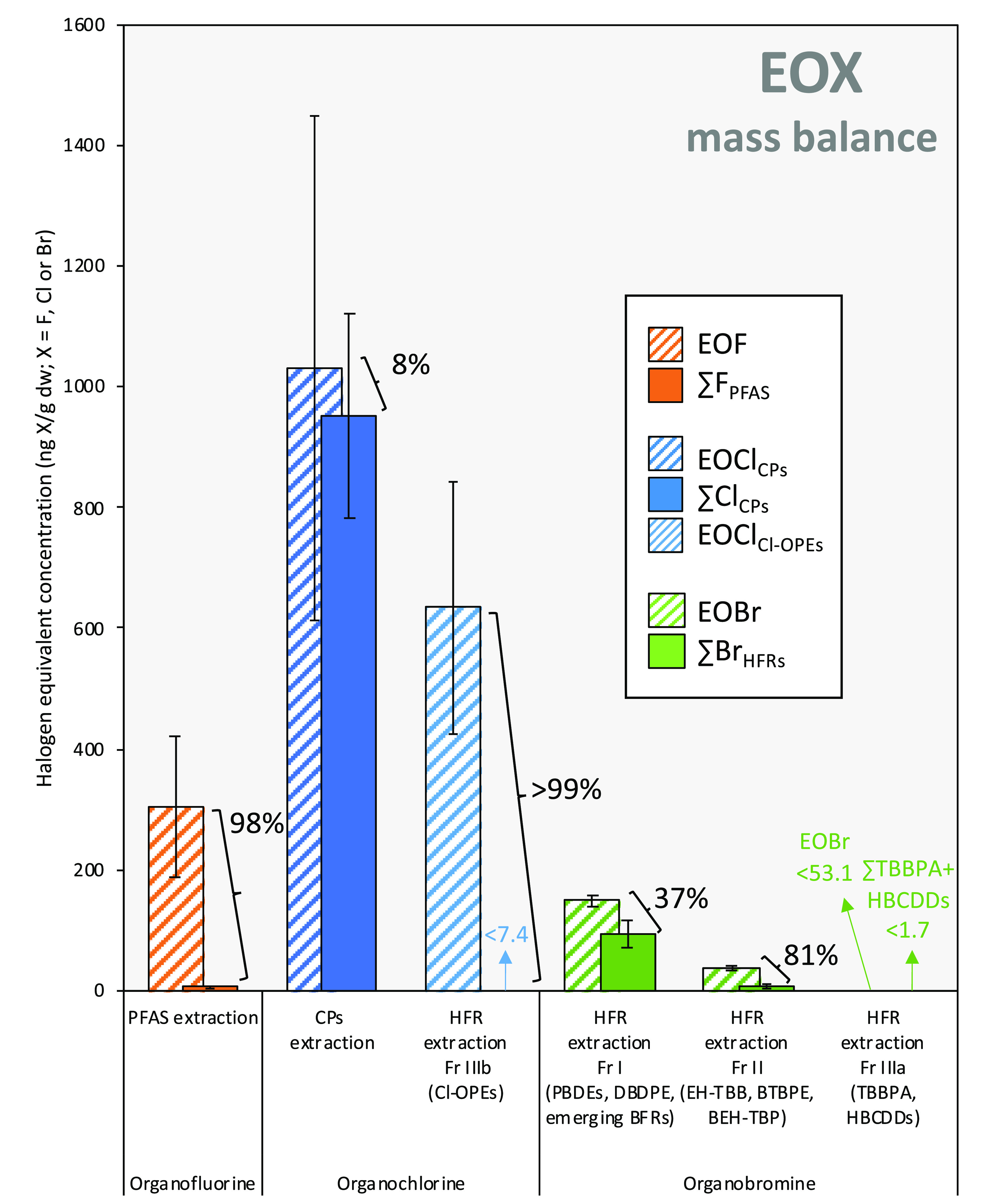
Extractable organofluorine, organochlorine, and organobromine mass balances in sewage sludge (*n* = 3). EOX concentrations from CIC are compared to the halogen equivalent concentrations from target analyses (nonrecovery corrected). Percentages show the size of the gap between identified and unidentified EOX. Error bars indicate standard deviation of replicate extractions. A detailed table including target analyte concentrations is presented in Table S16.

Six out of 16 target PFAS were detected in the sludge (Figure 3, Table S16). PFOS was the most prevalent target (7.2 ± 0.6 ng/g dw [sum isomers], recovery-corrected), which is consistent with prior measurements from the same location in 2019 and 2020 (7.7 and 11 ng/g dw, respectively).^30,33^ The sum fluorine equivalent concentration from target PFAS measured here (6.4 ± 1.0 ng F/g dw, nonrecovery corrected) accounted for a mere 2% of the 304 ± 116 ng F/g dw in sludge EOF (Figure 4), indicating that the vast majority of the organofluorine is unidentified. Previous reports of EOF concentrations in sludge from the same WWTP showed considerable variability, (i.e., 535–1270 ng F/g reported for samples from 2004 to 2015,^27^ 154 ng F/g from 2016,^31^ and ∼650 ng F/g from 2017^32^), albeit with slightly different procedures for extraction and quantification. For example, while all three studies used a similar methanolic extraction procedure, two included an alkaline digestion step, two used an ion-pair cleanup,^27,32^ and one used a weak anion exchange cleanup.^31^ Moreover, two were extraction-blank subtracted,^27,31^ and one was combustion-blank subtracted.^32^ This variability highlights the need for reference materials and a more standardized procedure.^26^ The report by Kärrman et al.^32^ analyzed sludge from the same WWTP and included an expanded target list of 78 PFAS, which despite the inclusion of more PFAS, could only characterize <10% of EOF. The main contributors of EOF in sludge were polyfluoroalkyl phosphate esters (PAPs), perfluooctane sulfonamidoacetates (FOSAAs), fluorotelomer carboxylic acids (FTCAs), and legacy PFCAs and PFSAs.^32^

Other possible contributors to EOF include fluorinated pharmaceuticals, pesticides, and polymers. Fluoropharmaceuticals and fluoropesticides typically only have a few fluorine atoms, but at high concentrations, these substances may contribute significantly to the fluorine mass balance. Roughly 20% of commercial pharmaceuticals contain one or more fluorine atoms.^47^ Fluorine has been introduced in active ingredients for crop protection during recent decades, and organofluorine represents a growing family of commercial agrochemicals.^48^ In fact, nearly 70% of all pesticides introduced into the global market from 2015 to 2020 contained fluorine.^49^ Another possibility are fluorinated polymers, which are known to be used throughout consumer products, including cosmetic products,^50^ and textiles.^51^ Letcher et al.^52^ and Fredriksson et al.^53^ previously observed side-chain fluorinated polymers in sewage sludge at elevated concentrations, but only by using a substantially different extraction procedure (i.e., acetone/hexane) than that employed here (MeOH). Thus, the contribution of fluorinated polymers to EOF determined here remains unclear.

#### CPs

MCCPs (1127 ± 203 ng/g dw) made up the vast majority (i.e., 62%), of CPs in sludge, followed by LCCPs (502 ± 114 ng/g dw; 28%) and SCCPs (181 ± 21 ng/g dw; 10%) (Figure 3, Table S16). Very short-chain CPs (C_6–9_H_*x*_Cl_*y*_) were also observed (5.5 ± 0.5 ng/g dw) but accounted for only 0.3% of the ∑CP concentration (Figure 3). The concentrations of SCCPs and MCCPs reported here aligned well with a recent report in Swedish sludge,^30^ and the congener profile from the present work was very similar to the profile of settled dust from Norway (Table S14).^38^ Prior CP measurements in Swedish sewage sludge between 2002 and 2010 have reported higher concentrations of SCCPs, MCCPs, and LCCPs (median concentrations were 1100, 3800, and 31000 ng/g dw for SCCPs, MCCPs, and LCCPs, respectively),^54^ possibly due to changes in production of specific chain lengths over the last 20 years,^55^ and/or differences in instrumental and quantification techniques.^56,57^

Conversion to chlorine equivalents revealed that ∑CP concentrations (951 ± 169 ng Cl/g dw) accounted for nearly all (i.e., 92%) of the measured EOCl_CP-extraction_ concentration (1030 ± 417 ng Cl/g; Figure 4). While the unidentified EOCl_CP-extraction_ was not statistically significant, we cannot rule out the occurrence of relevant organochlorine compounds which appear minor when considered on a chlorine equivalent basis, yet are much more significant when considering molar concentrations. Many pharmaceuticals, for example, although usually more polar and might not be present in this particular extract, contain chlorine atoms (even more so than fluorine and bromine^58^) and may occur at elevated molar concentrations in sludge. Furthermore, drinking water facilities use a small amount of chloramine for disinfection and could therefore contribute with chlorinated disinfection byproducts (DBPs).^59^

##### Chlorinated OPEs

Following blank subtraction, recovery-corrected TCIPP and TDCPP concentrations were 60.3 ± 8.7 and 4.2 ± 0.5 ng/g dw, respectively (Figure 3, Table S16). The EOCl_HFR-extraction_ for this fraction was 634 ± 208 ng Cl/g (Figure 4; Table 1). Due to low recoveries, the ∑Cl_Cl-OPEs_ of the nonrecovery-corrected fraction could not be calculated as concentrations from all three Cl-OPEs were below quantification limits. OPEs have been analyzed previously in Swedish WWTP sludge, and concentrations reported here are lower than measured before (see overview in Table S15).

##### BFRs

Among the analyzed BFRs, three compounds made up ∼90% of the known recovery-corrected ∑_35_BFR concentration: BDE209, DBDPE, and BEH-TEBP at 130 ± 10, 60 ± 7, and 30 ± 7 ng/g dw, respectively (Figure 3, Table S16). BDE209 is known to vary in WWTP sludge, with reported concentrations at Henriksdal ranging from 120 to 1760 ng/g dw from 2004 to 2020,^34^ ∼100–1050 ng/g dw in 2019,^30^ and 164–229 ng/g dw in 2020.^33^ EOBr mass balance for the different cleanup fractions resulted in 37% unidentified EOBr for Fraction I and 81% unidentified EOBr for Fraction II (Figure 4; Table 1). EOBr measurements were below detection limits in Fraction IIIa.

Possible sources of unidentified EOBr are numerous. For example, although more polar, hydroxylated, and methoxylated PBDEs (OH- and MeO-PBDEs) could end up in the same extracts as parent BDEs.^60^ Polybrominated dibenzo-p-dioxins and dibenzofurans (PBDD/Fs) can form after combustion of PBDEs.^61^ Another possible source could be from polymeric flame retardants.^62^ Brominated drugs, pesticides, rodenticides,^63^ and their metabolites could also contribute. Bromhexine, for example, is used in over-the-counter cough syrups and pastils, and bromocriptine, is used in the treatment of Parkinson’s. Both have been detected in influent, effluent, and sludge from Sweden.^64,65^ Several brominated herbicides are registered in the Swedish Chemical Agency’s pesticide register, e.g., bromacil and bromophos.^66^ However, these compounds are more polar and are less likely to be coextracted using the BFR extraction procedure employed here.

#### Prioritizing Extracts for Nontarget Investigation

Further characterization of samples investigated here using nontarget-based analytical approaches should prioritize extracts with both high EOX concentrations and large fractions of unidentified EOX. In the present work, PFAS extracts (304 ± 116 ng F/g dw EOF; 98% unknown) and HFR Fraction I (149 ± 10 ng Br/g dw EOBr; 37% unknown) and Fraction II (39 ± 4 ng Br/g dw EOBr; 81% unknown) extracts meet these criteria. Tools such as the total oxidizable precursors assay (TOP; for EOF),^67,68^ as well as high resolution mass spectrometry-based nontarget and suspect screening (for EOF and/or EOBr) could provide further insight into the unidentified fractions.^69^ With regards to chlorinated extracts, nearly all EOCl in the CP extract was accounted for by target CPs. For HFR Fractions II and IIIb, we expect that CPs also explain the vast majority of EOCl, despite that CPs were not measured in this sample, as they have been found to elute in similar fractions previously.^70^ Thus, on the basis of halogen equivalents, the HFR extracts from Fraction IIIb could be considered lower priority (albeit with some caveats, discussed in the following section).

In addition to the aforementioned halogen mass balances, we performed EOX measurements on several extracts where a particular halogen was unexpected (Table 1). For example, sludge CP extracts contained 54.0 ± 22 ng F/g dw, which was surprising considering the extraction procedure is designed to favor neutral, lipophilic substances, suggesting the presence of nonconventional PFAS or other fluorinated substances. Similarly, CP extracts from dust SRM 2585 contained 417 ± 77 ng F/g dw. There is a paucity of data on the occurrence of lipophilic organofluorine, with the exception of a single report of high EOF in the blubber of a Greenland Killer whale,^20^ and a more recent article predicting the environmental occurrence of mixed halogenated *n*-alkanes (including those containing fluorine).^71^ Similar observations for EOBr (e.g., 2340 ± 433 ng Br/g dw observed in CP extracts of SRM 2585) are also of interest but perhaps less remarkable since a wide range of lipophilic/nonpolar and lipophobic/polar organobromines are known. Thus, nontarget investigations of organofluorines in nonpolar CP or HFR extracts could be of considerable interest and worthwhile to prioritize for future investigations.

#### Perspectives and Limitations on the Use of OHC Mass Balance for Characterizing the OHC Iceberg

The present work represents the first multihalogen mass balance investigation in municipal WWTP sludge and offers unique insights into the extracts/extraction procedures which yield high concentrations of unidentified EOX. This approach offers a complementary tool to other nontarget prioritization strategies, such as case-control,^72^ time trends,^73^ and biomagnification factors,^74^ but is unique in that it identifies specific halogen class(es) and extraction technique(s), rather than a specific feature of a mass spectrum. However, despite showing considerable potential for widespread adoption and applications to a wider suite of matrices, there remains a scarcity of reference material necessary for standardization of halogen mass balance methods. While this work offers some of the first measurements of TX and EOX in publicly available reference materials, consensus values are needed across a much wider suite of matrices.

The halogen mass balance approach also has several limitations that researchers should be aware of prior to adopting it as a prioritization strategy. First and foremost, quantifying on a halogen-equivalent basis reduces the importance of substances with a low number of halogen atoms (or none at all), which may nevertheless exist at high concentrations and/or be important toxicologically. Halogenated pharmaceuticals, for example, are designed to be bioactive, yet typically only contain a few halogen atoms. In a similar vein, we cannot rule out that organohalogens occurring naturally in the terrestrial and marine environment (which may or may not be toxicologically relevant)^76^ may contribute to the halogen mass balance.

A fundamental limitation of halogen mass balance experiments is that EOX is method specific; i.e., different concentrations of EOX will be obtained using different methods since some relevant OHCs will be removed during extraction.^17^ For this reason, EOX should not be confused with “total” organic halogen, the latter of which can be conceptualized but is difficult to determine experimentally, since it requires assumptions about the method’s ability to extract all OHCs. While measurement of inorganic halogen concentrations and subtracting these from TX has been employed for “total” organohalogen determination in consumer products,^75^ this assumes that all inorganic halogens exist as halides (i.e., X^–^), which is unlikely for sludge. In comparison, EOX assumes that other inorganic halogens (if present) behave similarly to X^–^ and are removed during the extraction procedure. While it could be argued that a more holistic extraction method with fewer cleanup steps, or performing the halogen mass balance after each cleanup step, could capture a large fraction of the “total” organic halogen, these approaches would inevitably compromise the quality of targeted OHC data due to coextraction of matrix interferences. For these reasons, we argue that pairing EOX and targeted analyses on the same extract is the most appropriate and practical approach to performing a halogen mass balance.

## Supporting Information Available

Additional information for PFAS, CP, and HFR extraction procedures; schematic illustration of the WWTP; extraction workflow; HFR cleanup workflow; overview target PFAS, CPs, and HFRs; RTs, precursor, quantitative and qualitative ions, ISs, and LOQs for PFAS; spike/recovery results for PFAS, PFAS in NIST sludge material; LOQs for CPs, spike/recovery results for CPs; CPs in NIST dust material; RTs, precursor and quantitative ions, ISs and LOQs for HFRs; IS yields for HFRs; spike/recovery results for HFRs; eluent gradient for EOX analysis; results for NaF spike/recovery experiment; combustion efficiencies for CIC; CP homologue profile; OPE concentrations in literature; mass balance overview (PDF)

## References

[ref1] Wang Z.; Walker G. W.; Muir D. C. G.; Nagatani-Yoshida K. Toward a Global Understanding of Chemical Pollution: A First Comprehensive Analysis of National and Regional Chemical Inventories. Environ. Sci. Technol. 2020, 54, 2575 10.1021/acs.est.9b06379. 31968937

[ref2] Zou J. W.; Jiang Y. J.; Guo M.; Hu G. X.; Zhang B.; Liu H. C.; Yu Q. Sen. Ab Initio Study of the Complexes of Halogen-Containing Molecules RX (X = Cl, Br, and I) and NH3: Towards Understanding the Nature of Halogen Bonding and the Electron-Accepting Propensities of Covalently Bonded Halogen Atoms. Chem. - A Eur. J. 2005, 11 (2), 740–751. 10.1002/chem.200400504. 15584077

[ref3] Muir D.; Zhang X.; de Wit C. A.; Vorkamp K.; Wilson S. Identifying Further Chemicals of Emerging Arctic Concern Based on ‘in Silico’ Screening of Chemical Inventories. Emerg. Contam. 2019, 5, 201–210. 10.1016/j.emcon.2019.05.005.

[ref4] Waste Water Treatment - Sludge Management; EurEau, 2021.

[ref5] Kiceniuk J. W.; Holzbecher J.; Chatt A. Extractable Organohalogens in Tissues of Beluga Whales from the Canadian Arctic and the St. Lawrence Estuary. Environ. Pollut. 1997, 97 (3), 205–211. 10.1016/S0269-7491(97)00100-0. 15093357

[ref6] Kawano M.; Falandysz J.; Wakimoto T. Instrumental Neutron Activation Analysis of Extractable Organohalogens (EOX) in Antarctic Marine Organisms. J. Radioanal. Nucl. Chem. 2003, 255 (2), 235–237. 10.1023/A:1022563611414.

[ref7] Kawano M.; Falandysz J.; Wakimoto T. Instrumental Neutron Activation Analysis of Extractable Organohalogens in the Antarctic Weddell Seal (Leptonychotes Weddelli). J. Radioanal. Nucl. Chem. 2007, 272 (3), 501–504. 10.1007/s10967-007-0611-5.

[ref8] Kawano M.; Falandysz J.; Morita M. Instrumental Neutron Activation Analysis of Extractable Organohalogens in Marine Mammal, Harbour Porpoise (Phocoena Phocoena) and Its Feed, Atlantic Herring (Clupea Harengus), from the Baltic Sea. J. Radioanal. Nucl. Chem. 2008, 278 (2), 263–266. 10.1007/s10967-008-0401-8.

[ref9] Kannan K.; Kawano M.; Kashima Y.; Matsui M.; Giesy J. P. Extractable Organohalogens (EOX) in Sediment and Biota Collected at an Estuarine Marsh near a Former Chloralkali Facility. Environ. Sci. Technol. 1999, 33 (7), 1004–1008. 10.1021/es9811142.

[ref10] Loganathan B. G.; Kannan K.; Watanabe I.; Kawano M.; Irvine K.; Kumar S.; Sikka H. C. Isomer-Specific Determination and Toxic Evaluation of Polychlorinated Biphenyls, Polychlorinated/Brominated Dibenzo-p-Dioxins and Dibenzoftirans, Polybrominated Biphenyl Ethers, and Extractable Organic Halogen in Carp from the Buffalo River, New York. Environ. Sci. Technol. 1995, 29 (7), 1832–1838. 10.1021/es00007a021. 22176457

[ref11] Matsui M.; Kashima Y.; Kawano M.; Matsuda M.; Ambe K.; Wakimoto T.; Doi R. Dioxin-like Potencies and Extractable Organohalogens (EOX) in Medical, Municipal and Domestic Waste Incinerator Ashes in Japan. Chemosphere 2003, 53 (8), 971–980. 10.1016/S0045-6535(03)00587-3. 14505720

[ref12] Xu D.; Dan M.; Song Y.; Chai Z.; Zhuang G. Concentration Characteristics of Extractable Organohalogens in PM2.5 and PM10 in Beijing, China. Atmos. Environ. 2005, 39 (22), 4119–4128. 10.1016/j.atmosenv.2005.03.030.

[ref13] Xu D.; Zhong W.; Deng L.; Chai Z.; Mao X. Levels of Extractable Organohalogens in Pine Needles in China. Environ. Sci. Technol. 2003, 37 (1), 1–6. 10.1021/es025799o. 12542283

[ref14] Mukai K.; Fujimori T.; Anh H. Q.; Fukutani S.; Kunisue T.; Nomiyama K.; Takahashi S. Extractable Organochlorine (EOCl) and Extractable Organobromine (EOBr) in GPC-Fractionated Extracts from High-Trophic-Level Mammals: Species-Specific Profiles and Contributions of Legacy Organohalogen Contaminants. Sci. Total Environ. 2021, 756, 143843 10.1016/j.scitotenv.2020.143843. 33303197

[ref15] Miyake Y.; Yamashita N.; Rostkowski P.; So M. K.; Taniyasu S.; Lam P. K. S.; Kannan K. Determination of Trace Levels of Total Fluorine in Water Using Combustion Ion Chromatography for Fluorine: A Mass Balance Approach to Determine Individual Perfluorinated Chemicals in Water. J. Chromatogr. A 2007, 1143 (1–2), 98–104. 10.1016/j.chroma.2006.12.071. 17229428

[ref16] Miyake Y.; Yamashita N.; So M. K.; Rostkowski P.; Taniyasu S.; Lam P. K. S.; Kannan K. Trace Analysis of Total Fluorine in Human Blood Using Combustion Ion Chromatography for Fluorine: A Mass Balance Approach for the Determination of Known and Unknown Organofluorine Compounds. J. Chromatogr. A 2007, 1154 (1–2), 214–221. 10.1016/j.chroma.2007.03.084. 17416376

[ref17] Kaiser A. M.; Aro R.; Kärrman A.; Weiss S.; Hartmann C.; Uhl M.; Forsthuber M.; Gundacker C.; Yeung L. W. Y. Comparison of Extraction Methods for Per- and Polyfluoroalkyl Substances (PFAS) in Human Serum and Placenta Samples—Insights into Extractable Organic Fluorine (EOF). Anal. Bioanal. Chem. 2021, 413, 865–876. 10.1007/s00216-020-03041-5. 33215313 PMC7809006

[ref18] Koch A.; Kärrman A.; Yeung L. W. Y.; Jonsson M.; Ahrens L.; Wang T. Point Source Characterization of Per- and Polyfluoroalkyl Substances (PFASs) and Extractable Organofluorine (EOF) in Freshwater and Aquatic Invertebrates. Environ. Sci. Process. Impacts 2019, 21, 1887 10.1039/C9EM00281B. 31552402

[ref19] Miaz L. T.; Plassmann M. M.; Gyllenhammar I.; Bignert A.; Sandblom O.; Lignell S.; Glynn A.; Benskin J. P. Temporal Trends of Suspect-and Target-per/Polyfluoroalkyl Substances (PFAS), Extractable Organic Fluorine (EOF) and Total Fluorine (TF) in Pooled Serum from First-Time Mothers in Uppsala, Sweden, 1996–2017. Environ. Sci. Process. Impacts 2020, 22 (4), 1071–1083. 10.1039/C9EM00502A. 32182307

[ref20] Schultes L.; Van Noordenburg C.; Spaan K. M.; Plassmann M. M.; Simon M.; Roos A.; Benskin J. P. High Concentrations of Unidentified Extractable Organofluorine Observed in Blubber from a Greenland Killer Whale (Orcinus Orca). Environ. Sci. Technol. Lett. 2020, 7, 909 10.1021/acs.estlett.0c00661.

[ref21] Spaan K. M.; van Noordenburg C.; Plassmann M. M.; Schultes L.; Shaw S.; Berger M.; Heide-Jørgensen M. P.; Rosing-Asvid A.; Granquist S. M.; Dietz R.; Sonne C.; Rigét F.; Roos A.; Benskin J. P. Fluorine Mass Balance and Suspect Screening in Marine Mammals from the Northern Hemisphere. Environ. Sci. Technol. 2020, 54, 4046 10.1021/acs.est.9b06773. 32160740 PMC7309329

[ref22] Yeung L. W. Y.; Miyake Y.; Wang Y.; Taniyasu S.; Yamashita N.; Lam P. K. S. Total Fluorine, Extractable Organic Fluorine, Perfluorooctane Sulfonate and Other Related Fluorochemicals in Liver of Indo-Pacific Humpback Dolphins (Sousa Chinensis) and Finless Porpoises (Neophocaena Phocaenoides) from South China. Environ. Pollut. 2009, 157 (1), 17–23. 10.1016/j.envpol.2008.08.005. 18805607

[ref23] Schultes L.; Vestergren R.; Volkova K.; Westberg E.; Jacobson T.; Benskin J. P. Per- and Polyfluoroalkyl Substances and Fluorine Mass Balance in Cosmetic Products from the Swedish Market: Implications for Environmental Emissions and Human Exposure. Environ. Sci. Process. Impacts 2018, 20, 1680 10.1039/C8EM00368H. 30427048

[ref24] Schultes L.; Peaslee G. F.; Brockman J. D.; Majumdar A.; McGuinness S. R.; Wilkinson J. T.; Sandblom O.; Ngwenyama R. A.; Benskin J. P. Total Fluorine Measurements in Food Packaging: How Do Current Methods Perform?. Environ. Sci. Technol. Lett. 2019, 6, 73–78. 10.1021/acs.estlett.8b00700.

[ref25] Aro R.; Eriksson U.; Kärrman A.; Chen F.; Wang T.; Yeung L. W. Y. Fluorine Mass Balance Analysis of Effluent and Sludge from Nordic Countries. ACS ES&T Water 2021, 1 (9), 2087–2096. 10.1021/acsestwater.1c00168.

[ref26] Kärrman A.; Yeung L. W. Y.; Spaan K. M.; Lange F. T.; Nguyen M. A.; Plassmann M.; de Wit C. A.; Scheurer M.; Awad R.; Benskin J. P. Can Determination of Extractable Organofluorine (EOF) Be Standardized? First Interlaboratory Comparisons of EOF and Fluorine Mass Balance in Sludge and Water Matrices. Environ. Sci. Process. Impacts 2021, 23, 1458 10.1039/D1EM00224D. 34546240

[ref27] Yeung L.; Eriksson U.; Kärrman A. Time Trend of Unidentified Poly- and Perfluoroalkyl Substances in Sludge from Wastewater Treatment Plants in Sweden, 2017. National Environmental Monitoring Report Commisioned by the Swedish EPA. http://naturvardsverket.diva-portal.org/smash/record.jsf?pid=diva2%3A1138488&dswid=-9656 (accessed March 6, 2023).

[ref28] Miljörapport 2019 - Avloppsverksamenheten Vid Stockholm Vatten Och Avfall; SVOA: Stockholm, 2019.

[ref29] van Leeuwen C. J.; Vermeire T. G. Risk Assessment of Chemicals: An Introduction, Second ed.; Springer: Dordrecht, The Netherlands, 2007. 10.2166/9781789061987_0025.

[ref30] Närhi K.; Westling K.; Andersson S.; Baresel C.; Wahlberg C. Mikroföroreningar i Avloppsreningsverk Med. Membranteknik; rapport nr 2021-2, Svenskt Vatten, 2021; ISSN: 1654-644X. https://vattenbokhandeln.svensktvatten.se/wp-content/uploads/2021/06/svu-rapport-2021-02.pdf (accessed March 6, 2023).

[ref31] Yeung L. W. Y.; Eriksson U.; Kärrman A. Pilotstudie Avseende Oidentifierade Poly- Och Perfluorerade Alkylämnen i Slam Och Avloppsvatten Från Reningsverk i Sverige, 2016. National Environmental Monitoring Report Commisioned by the Swedish EPA. http://naturvardsverket.diva-portal.org/smash/record.jsf?pid=diva2%3A1112388&dswid=-158 (accessed March 6, 2023).

[ref32] Kärrman A.; Wang T.; Kallenborn R.; Langseter A. M.; Grønhovd S. M.; Ræder E. M.; Lyche J. L.; Yeung L.; Chen F.; Eriksson U.; Aro R.; Fredriksson F. PFASs in the Nordic Environment; Nordic Council of Ministers, 2019.

[ref33] Miljörapport 2020; SVOA: Stockholm, 2020.

[ref34] BDE209 i rötslam (DekaBDE). Stockholms Stad. https://miljobarometern.stockholm.se/miljogifter/bromerade-flamskyddsmedel/bde209-i-rotslam/henriksdal/table/ (accessed Feb 1, 2022).

[ref35] Powley C. R.; George S. W.; Ryan T. W.; Buck R. C. Matrix Effect-Free Analytical Methods for Determination of Perfluorinated Carboxylic Acids in Environmental Matrixes. Anal. Chem. 2005, 77 (19), 6353–6358. 10.1021/ac0508090. 16194099

[ref36] Yuan B.; Wang Y. W.; Fu J. J.; Zhang Q. H.; Jiang G. Bin. An Analytical Method for Chlorinated Paraffins and Their Determination in Soil Samples. Chin. Sci. Bull. 2010, 55 (22), 2396–2402. 10.1007/s11434-010-3261-x.

[ref37] Yuan B.; Rüdel H.; de Wit C. A.; Koschorreck J. Identifying Emerging Environmental Concerns from Long-Chain Chlorinated Paraffins towards German Ecosystems. J. Hazard. Mater. 2022, 424 (PC), 127607 10.1016/j.jhazmat.2021.127607. 34768030

[ref38] Yuan B.; Tay J. H.; Padilla-Sánchez J. A.; Papadopoulou E.; Haug L. S.; De Wit C. A. Human Exposure to Chlorinated Paraffins via Inhalation and Dust Ingestion in a Norwegian Cohort. Environ. Sci. Technol. 2021, 55 (2), 1145–1154. 10.1021/acs.est.0c05891. 33400865 PMC7880561

[ref39] Bogdal C.; Alsberg T.; Diefenbacher P. S.; Macleod M.; Berger U. Fast Quantification of Chlorinated Paraffins in Environmental Samples by Direct Injection High-Resolution Mass Spectrometry with Pattern Deconvolution. Anal. Chem. 2015, 87 (5), 2852–2860. 10.1021/ac504444d. 25668073

[ref40] Nylund K.; Asplund L.; Jansson B.; Jonsson P.; Litzen K.; Sellström U. Analysis of Some Polyhalogenated Organic Pollutants in Sediment and Sewage Sludge. Chemosphere 1992, 24, 1721–1730. 10.1016/0045-6535(92)90227-I.

[ref41] Sahlström L.; Sellström U.; De Wit C. A. Clean-up Method for Determination of Established and Emerging Brominated Flame Retardants in Dust. Anal. Bioanal. Chem. 2012, 404 (2), 459–466. 10.1007/s00216-012-6160-y. 22692590

[ref42] Hites R. A. Polybrominated Diphenyl Ethers in the Environment and in People: A Meta-Analysis of Concentrations. Environ. Sci. Technol. 2004, 38 (4), 945–956. 10.1021/es035082g. 14998004

[ref43] Shang H.; Fan X.; Kubwabo C.; Rasmussen P. E. Short-Chain and Medium-Chain Chlorinated Paraffins in Canadian House Dust and NIST SRM 2585. Environ. Sci. Pollut. Res. 2019, 26 (8), 7453–7462. 10.1007/s11356-018-04073-2. 30656583

[ref44] Brits M.; de Boer J.; Rohwer E. R.; De Vos J.; Weiss J. M.; Brandsma S. H. Short-, Medium-, and Long-Chain Chlorinated Paraffins in South African Indoor Dust and Cat Hair. Chemosphere 2020, 238, 124643 10.1016/j.chemosphere.2019.124643. 31473532

[ref45] Brandsma S. H.; Brits M.; Groenewoud Q. R.; Van Velzen M. J. M.; Leonards P. E. G.; De Boer J. Chlorinated Paraffins in Car Tires Recycled to Rubber Granulates and Playground Tiles. Environ. Sci. Technol. 2019, 53 (13), 7595–7603. 10.1021/acs.est.9b01835. 31181880 PMC6610544

[ref46] Aro R.; Eriksson U.; Karrman A.; Reber I.; Yeung L. W.Y. Combustion Ion Chromatography for Extractable Organofluorine Analysis. iScience 2021, 24, 102968 10.1016/j.isci.2021.102968. 34466791 PMC8383001

[ref47] Inoue M.; Sumii Y.; Shibata N. Contribution of Organofluorine Compounds to Pharmaceuticals. ACS Omega 2020, 5 (19), 10633–10640. 10.1021/acsomega.0c00830. 32455181 PMC7240833

[ref48] Jeschke P. The Unique Role of Fluorine in the Design of Active Ingredients for Modern Crop Protection. ChemBioChem. 2004, 5 (5), 570–589. 10.1002/cbic.200300833. 15122630

[ref49] Alexandrino D. A. M.; Almeida C. M. R.; Mucha A. P.; Carvalho M. F. Revisiting Pesticide Pollution: The Case of Fluorinated Pesticides. Environ. Pollut. 2022, 292 (PA), 118315 10.1016/j.envpol.2021.118315. 34634397

[ref50] Pütz K. W.; Namazkar S.; Plassmann M.; Benskin J. P. Are Cosmetics a Significant Source of PFAS in Europe? Product Inventories, Chemical Characterization and Emission Estimates. Environ. Sci. Process. Impacts 2022, 24, 1697 10.1039/D2EM00123C. 35959763

[ref51] Schellenberger S.; Jönsson C.; Mellin P.; Levenstam O. A.; Liagkouridis I.; Ribbenstedt A.; Hanning A. C.; Schultes L.; Plassmann M. M.; Persson C.; Cousins I. T.; Benskin J. P. Release of Side-Chain Fluorinated Polymer-Containing Microplastic Fibers from Functional Textiles during Washing and First Estimates of Perfluoroalkyl Acid Emissions. Environ. Sci. Technol. 2019, 53 (24), 14329–14338. 10.1021/acs.est.9b04165. 31697071

[ref52] Letcher R. J.; Chu S.; Smyth S. A. Side-Chain Fluorinated Polymer Surfactants in Biosolids from Wastewater Treatment Plants. J. Hazard. Mater. 2020, 388, 122044 10.1016/j.jhazmat.2020.122044. 31955025

[ref53] Fredriksson F.; Eriksson U.; Kärrman A.; Yeung L. W. Y. Per- and Polyfluoroalkyl Substances (PFAS) in Sludge from Wastewater Treatment Plants in Sweden — First Findings of Novel Fluorinated Copolymers in Europe Including Temporal Analysis. Sci. Total Environ. 2022, 846 (July), 157406 10.1016/j.scitotenv.2022.157406. 35850346

[ref54] Olofsson U.; Bignert A.; Haglund P. Time-Trends of Metals and Organic Contaminants in Sewage Sludge. Water Res. 2012, 46 (15), 4841–4851. 10.1016/j.watres.2012.05.048. 22763293

[ref55] Yuan B.; Brüchert V.; Sobek A.; De Wit C. A. Temporal Trends of C8-C36 Chlorinated Paraffins in Swedish Coastal Sediment Cores over the Past 80 Years. Environ. Sci. Technol. 2017, 51 (24), 14199–14208. 10.1021/acs.est.7b04523. 29155575

[ref56] Fernandes A. R.; Vetter W.; Dirks C.; van Mourik L.; Cariou R.; Sprengel J.; Heeb N.; Lentjes A.; Krätschmer K. Determination of Chlorinated Paraffins (CPs): Analytical Conundrums and the Pressing Need for Reliable and Relevant Standards. Chemosphere 2022, 286, 131878 10.1016/j.chemosphere.2021.131878. 34416588

[ref57] van Mourik L. M.; van der Veen I.; Crum S.; de Boer J. Developments and Interlaboratory Study of the Analysis of Short-Chain Chlorinated Paraffins. TrAC - Trends Anal. Chem. 2018, 102, 32–40. 10.1016/j.trac.2018.01.004.

[ref58] Smith B. R.; Eastman C. M.; Njardarson J. T. Beyond CHO, and N! Analysis of the Elemental Composition of U.S. FDA Approved Drug Architectures. J. Med. Chem. 2014, 57 (23), 9764–9773. 10.1021/jm501105n. 25255063

[ref59] Andersson A.; Harir M.; Gonsior M.; Hertkorn N.; Schmitt-Kopplin P.; Kylin H.; Karlsson S.; Ashiq M. J.; Lavonen E.; Nilsson K.; Pettersson A.; Stavklint H.; Bastviken D. Waterworks-Specific Composition of Drinking Water Disinfection by-Products. Environ. Sci. Water Res. Technol. 2019, 5 (5), 861–872. 10.1039/C9EW00034H.

[ref60] Kim U. J.; Oh J. E. Mass-Flow-Based Removal and Transformation Potentials for TBBPA, HBCDs and PBDEs during Wastewater Treatment Processes. J. Hazard. Mater. 2018, 355 (April), 82–88. 10.1016/j.jhazmat.2018.04.011. 29775881

[ref61] Zhang M.; Buekens A.; Li X. Brominated Flame Retardants and the Formation of Dioxins and Furans in Fires and Combustion. J. Hazard. Mater. 2016, 304, 26–39. 10.1016/j.jhazmat.2015.10.014. 26546701

[ref62] Minet L.; Blum A.; Fernandez S. R.; Rodgers K. M.; Singla V.; Soehl A.; Diamond M. L. High Production, Low Information: We Need To Know More About Polymeric Flame Retardants ’. Environ. Sci. Technol. 2021, 55, 3467–3469. 10.1021/acs.est.0c08126. 33618514

[ref63] Gómez-Canela C.; Lacorte S. Comprehensive Characterization of Anticoagulant Rodenticides in Sludge by Liquid Chromatography-Tandem Mass Spectrometry. Environ. Sci. Pollut. Res. 2016, 23 (15), 15739–15748. 10.1007/s11356-016-6743-9. 27146526

[ref64] Wahlberg C.; Björlenius B.; Paxéus N. Läkemedelsrester i Stockholms Vattenmiljö; Stockholm Vatten, 2010.

[ref65] Fick J.; Lindberg R. H.; Kaj L.; Brorström-Lundén E. Results from the Swedish National Screening Programme 2010; Swedish Environmental Research Institute, 2011.

[ref66] Bekämpningsmedelsregistret, KemI. https://apps.kemi.se/BkmRegistret/Kemi.Spider.Web.External/ (accessed Jun 2, 2022).

[ref67] Hutchinson S.; Rieck T.; Wu X. L. Advanced PFAS Precursor Digestion Methods for Biosolids. Environ. Chem. 2020, 17 (8), 558–567. 10.1071/EN20008.

[ref68] Houtz E. F.; Sedlak D. L. Oxidative Conversion as a Means of Detecting Precursors to Perfluoroalkyl Acids in Urban Runoff. Environ. Sci. Technol. 2012, 46 (17), 9342–9349. 10.1021/es302274g. 22900587

[ref69] González-Gaya B.; Lopez-Herguedas N.; Bilbao D.; Mijangos L.; Iker A. M.; Etxebarria N.; Irazola M.; Prieto A.; Olivares M.; Zuloaga O. Suspect and Non-Target Screening: The Last Frontier in Environmental Analysis. Anal. Methods 2021, 13 (16), 1876–1904. 10.1039/D1AY00111F. 33913946

[ref70] Tomy G. T.; Stern G. A.; Muir D. C. G.; Fisk A. T.; Cymbalisty C. D.; Westmore J. B. Quantifying C10-C13 Polychloroalkanes in Environmental Samples by High-Resolution Gas Chromatography/Electron Capture Negative Ion High-Resolution Mass Spectrometry. Anal. Chem. 1997, 69 (14), 2762–2771. 10.1021/ac961244y.

[ref71] Li X.; Chevez T.; De Silva A. O.; Muir D. C. G.; Kleywegt S.; Simpson A.; Simpson M. J.; Jobst K. J. Which of the (Mixed) Halogenated n-Alkanes Are Likely to Be Persistent Organic Pollutants?. Environ. Sci. Technol. 2021, 55, 15912 10.1021/acs.est.1c05465. 34802231

[ref72] Rotander A.; Kärrman A.; Toms L. M. L.; Kay M.; Mueller J. F.; Gómez Ramos M. J. Novel Fluorinated Surfactants Tentatively Identified in Firefighters Using Liquid Chromatography Quadrupole Time-of-Flight Tandem Mass Spectrometry and a Case-Control Approach. Environ. Sci. Technol. 2015, 49 (4), 2434–2442. 10.1021/es503653n. 25611076

[ref73] Plassmann M. M.; Tengstrand E.; Åberg K. M.; Benskin J. P. Non-Target Time Trend Screening : A Data Reduction Strategy for Detecting Emerging Contaminants in Biological Samples. Anal. Bioanal. Chem. 2016, 408, 4203–4208. 10.1007/s00216-016-9563-3. 27117254 PMC4875932

[ref74] Haglund P.; Rebryk A. Biomagnification and Temporal Trends of New and Emerging Dechloranes and Related Transformation Products in Baltic Sea Biota. Environ. Sci. Technol. Lett. 2022, 9, 406 10.1021/acs.estlett.2c00171. 35573270 PMC9097483

[ref75] Herkert N. J.; Kassotis C. D.; Zhang S.; Han Y.; Pulikkal V. F.; Sun M.; Ferguson P. L.; Stapleton H. M. Characterization of Per- and Polyfluorinated Alkyl Substances Present in Commercial Anti-Fog Products and Their In Vitro Adipogenic Activity. Environ. Sci. Technol. 2022, 56, 1162 10.1021/acs.est.1c06990. 34985261 PMC8908479

[ref76] Gribble G. W. A Recent Survey of Naturally Occurring Organohalogen Compounds. Environ. Chem. 2015, 12 (4), 396–405. 10.1071/EN15002.

