# Supplementary material for: Characterizing the Organohalogen Iceberg: Extractable, Multihalogen Mass Balance Determination in Municipal Wastewater Treatment Plant Sludge
Source: Environ Sci Technol. 2023 Jun 12;57(25):9309–20. doi: 10.1021/acs.est.3c01212 (PMC10308827; doi:10.1021/acs.est.3c01212)
Supplement: Supplementary file 1 — es3c01212_si_001.pdf [file es3c01212_si_001.pdf]

Supporting information

Characterizing the Organohalogen Iceberg: Extractable, multi-halogen mass balance determination in municipal wastewater treatment plant sludge

Kyra M. Spaan[1*], Bo Yuan[1,2], Merle M. Plassmann[1], Jonathan P. Benskin[1*], Cynthia A. de Wit[1*]
[1]Department of Environmental Science, Stockholm University, Svante Arrhenius väg 8, SE-106 91 Stockholm, Sweden
[2]Department of Chemistry, Norwegian University of Science and Technology, Høgskoleringen 5, N-7491 Trondheim, Norway

*Corresponding authors: Kyra.Spaan@aces.su.se; Jon.Benskin@aces.su.se; Cynthia.deWit@aces.su.se

Contents: 3 Figures; 16 tables; 16 pages

Table of Contents

Supplementary text
PFAS extraction procedure
CP extraction procedure
HFR extraction procedure
Supplementary figures
Figure S1. Schematic illustration of Henriksdal WWTP
Figure S2. Extraction workflow overview
Figure S3. Clean-up workflow for HFRs
Supplementary tables
Table S1. Target PFAS, CPs, and HFRs
Table S2. Target PFAS with RTs, precursor, quantitative and qualitative ions, ISs, and LOQs
Table S3. Spike/recovery results for PFAS
Table S4. PFAS in NIST sludge
Table S5. LOQs for CPs
Table S6. Spike/recovery results for CPs
Table S7. CPs in NIST dust
Table S8. Target HFRs with RTs, precursor and quantitative ions, ISs, and LOQs
Table S9. IS yields for HFRs
Table S10. Spike/recovery results for HFRs
Table S11. Eluent gradient for EOX analysis
Table S12. Results NaF spike/recovery experiment
Table S13. Combustion efficiencies for organic standard solutions for CIC
Table S14. CPs homologue profiles
Table S15. Concentrations of OPEs in sludge from literature
Table S16. Mass balance overview

Supplementary text

PFAS extraction procedure. Briefly, for extract 1 (Figure 1A) about 0.5 g freeze-dried sludge was weighed and fortified with internal standards. After extraction with methanol, the sample was vortexed, sonicated, and centrifuged. The supernatant was transferred to a clean centrifuge tube, and the extraction was repeated. The extract was reduced under nitrogen to a final volume of 1 mL, fortified with EnviCarb, and finally vortexed and centrifuged. The resulting extract was stored for instrumental analysis and the concentrations generated from this analysis were used for displaying PFAS profiles. The procedure was the same for fluorine mass balance analysis (Extract 2; Figure 1A), but internal standards were not added prior to extraction. Following EnviCarb clean-up 550 µL of extract was removed for EOF analysis and 50 µL was removed and fortified with 50 µL aqueous ammonium acetate buffer, and 5 µL of 200 pg/µL IS mix (in methanol) for targeted analysis.

CP extraction procedure. Accelerated solvent extraction (ASE) was performed on an ASE350 (Dionex, US) based on the method by Yuan et al.[1] All glassware used in sample extraction and clean-up was burned at 450 °C for >8 hours before use. ASE cells were pre-cleaned using the instrument. Diatomaceous earth (DE; Isolute HM-N, Biotage, UK) was used as a dispersing agent and was pre-cleaned using the same ASE method as for the samples (described below), after which it was burned at 450 °C in a muffle furnace for 6 hours. The cleaned ASE cells were packed with a glass fiber filter, a thin layer of pre-cleaned DE, ~0.5 g freeze-dried sludge, and more DE to pack the cell to nearly full. Extraction was performed at 100 °C and 1500 psi with thermal equilibration time of 5 min, and static extractions of 10 min using two extraction cycles. The cell was purged with gaseous nitrogen for 100 s and n-hexane:acetone (1:1) was used as the extraction solvent.

The extract was evaporated to near dryness with nitrogen and underwent three clean-up steps. i) Lipid removal: after solvent exchange to ~2 mL hexane, the extract was treated with ~6 mL concentrated sulfuric acid (98%, AnalaR, VWR, US). The leak-tested tube was rocked 15-20 times and centrifuged at 2000 rpm for 10 min. The organic phase was collected in a new tube, more hexane was added to the tube with acid, and the treatment was repeated. The extract was reduced to 2-3 mL. ii) Elemental sulfur removal: copper powder (~12 g; <63 µm, Merck kGaA, Darmstadt) was activated using concentrated hydrochloric acid (37%, AnalaR, VWR, France, diluted to 6 M) and then rinsed thoroughly with water (10×70 mL), methanol (10×70 mL), and acetone (10×70 mL) before adding to the extracts. Direct CIC analysis of the copper after rinsing (n=3) confirmed that the hydrochloric acid had been removed efficiently as concentrations were below the detection limit. iii) Clean-up: a multilayer solid phase extraction (SPE) column (Biotage, 15 mL with frit) was packed with 2 g silica (0.063-0.200 mm, Merck, Darmstadt; deactivated with 2.5% water), 1 g dried sodium sulfate (Honeywell Fluka), and a frit. The column was washed and conditioned with 20 mL n-hexane:acetone (1:1). The extract was added onto the column and eluted using 10 mL n-hexane:acetone (1:1). The final extract was concentrated to ~1 mL. An aliquot of 150 µL was spiked with 10 ng ^{13}C-labeled $C_{10}Cl_6$ and used for target analysis. The rest of the extract was analyzed for EOCl content using CIC.

HFR extraction procedure. For HFR analysis, a liquid-liquid extraction was performed based on a previous method by Nylund et al.[2] All glassware used in sample extraction and clean-up was burned at 450 °C for >8 hours before use. Approximately 2 g freeze-dried sludge was weighed and transferred into a tube with 40 mL acetone, which was rotated for 60 minutes and centrifuged. The liquid phase was transferred to a separatory funnel containing 50 mL buffer solution (0.9% NaCl in 0.1 M phosphorous acid). The solid phase was extracted again with a mixture of 10 mL acetone, 30 mL *n*-

hexane which was rotated 30 minutes and centrifuged. The liquid phase was added to the separatory funnel. After gentle rocking, the organic phase was collected and the buffer was re-extracted with 10 mL *n*-hexane:diethyl ether (9:1). The extract was concentrated under gentle nitrogen flow and the solvent was exchanged to isooctane.

Fractionation of the extract was performed using a silica column based on a method previously described by Sahlström et al.[3] with slight modifications. In short, a single fritted column (Isolute 15 mL 20 µm PE, Biotage) was packed with 2 g deactivated silica gel (0.063-0.200 mm, Merck), 1 g dried sodium sulfate and a frit. After washing the column with 40 mL *n*-hexane, the extract was added. The analytes were eluted in three fractions that were subsequently cleaned up individually (Figure S2) and in addition to targeted analysis also EOX was determined. Fraction I contained PBDEs, decabromodiphenyl ethane (DBDPE), and some emerging BFRs, fraction II included 2-ethylhexyl 2,3,4,5-tetrabromobenzoate (EH-TBB), 1,2-bis(2,4,6-tribromophenoxy)ethane (BTBPE), and bis(2-ethylhexyl) tetrabromophthalate (BEH-TEBP). Fraction IIIa contained tetrabromobisphenol A (TBBPA), α- β-, and γ- 1,2,5,6,9,10-hexabromocyclododecane (HBCDD), and fraction IIIb contained chlorinated OPEs. Fraction I and II were analyzed on a gas chromatography mass spectrometer (GC-MS) equipped with an electron capture negative ionization (ECNI) source. Since highly brominated PBDEs are prone to thermal degradation,[4] the octaBDEs, nonaBDEs and decaBDEs were analyzed using a short column (15 m), while the remaining BDEs were analyzed on a 30 m column. Fr IIIa was run on a LC-MS/ESI. Fr IIIb analysis was carried out on a GC-MS coupled to an electron ionization (GC-MS/EI), see Sahlström et al.[3] for instrumental details. Retention times and LOQs are available in the SI (Table S8). IS yields in blanks and sludge samples are presented in Table S9. For ISs with recoveries below 20% or above 150% (for TBBPA, HBCDDs, and TCEP), no quantification was performed. Accuracy and precision were evaluated using replicate (*n*=3) spike/recovery experiments performed at concentrations ~5 times higher than the measured/expected concentration in sludge (Table S10).

Supplementary figures

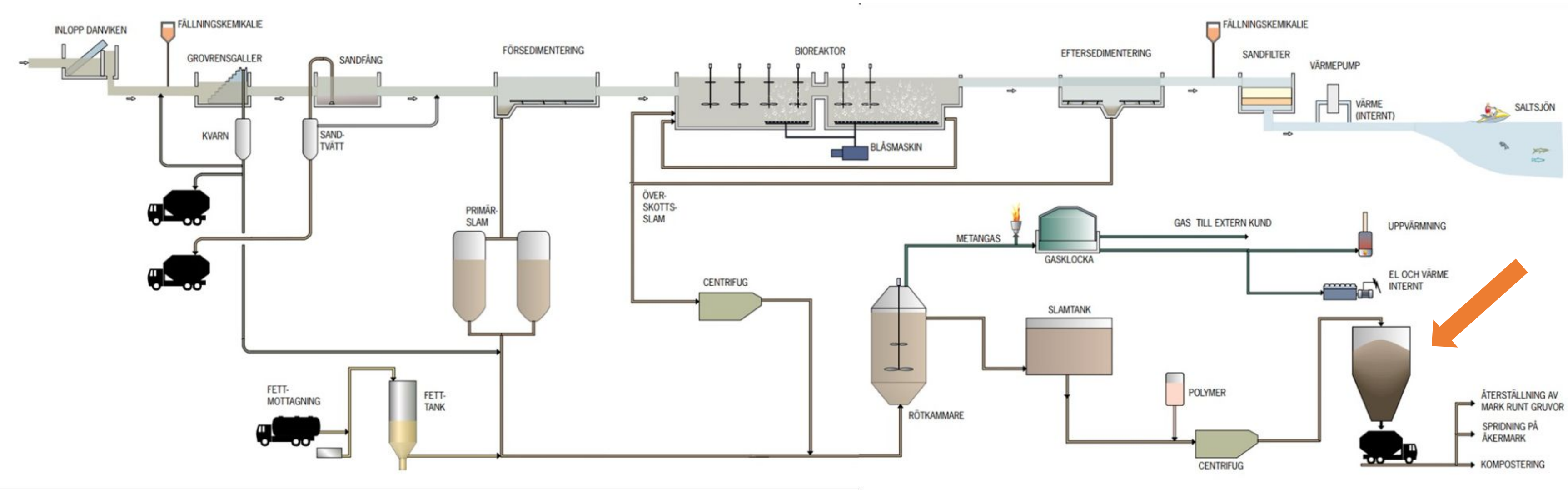

Figure S1. Schematic illustration of Henriksdal WWTP (figure adapted from Henriksdal's brochure[5]). Orange arrow indicates sampling location.

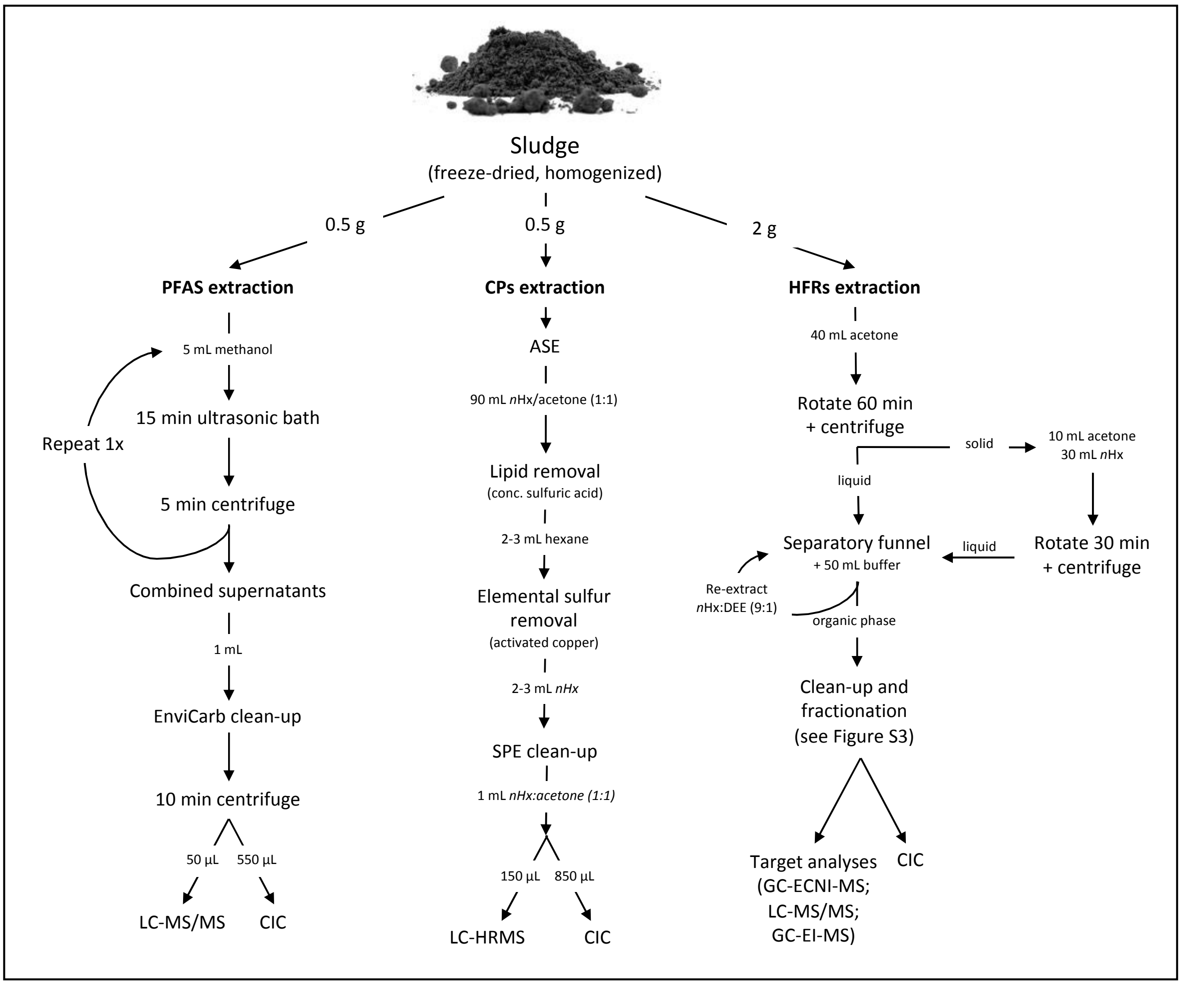

Figure S2. Schematic illustration of the extraction workflow.

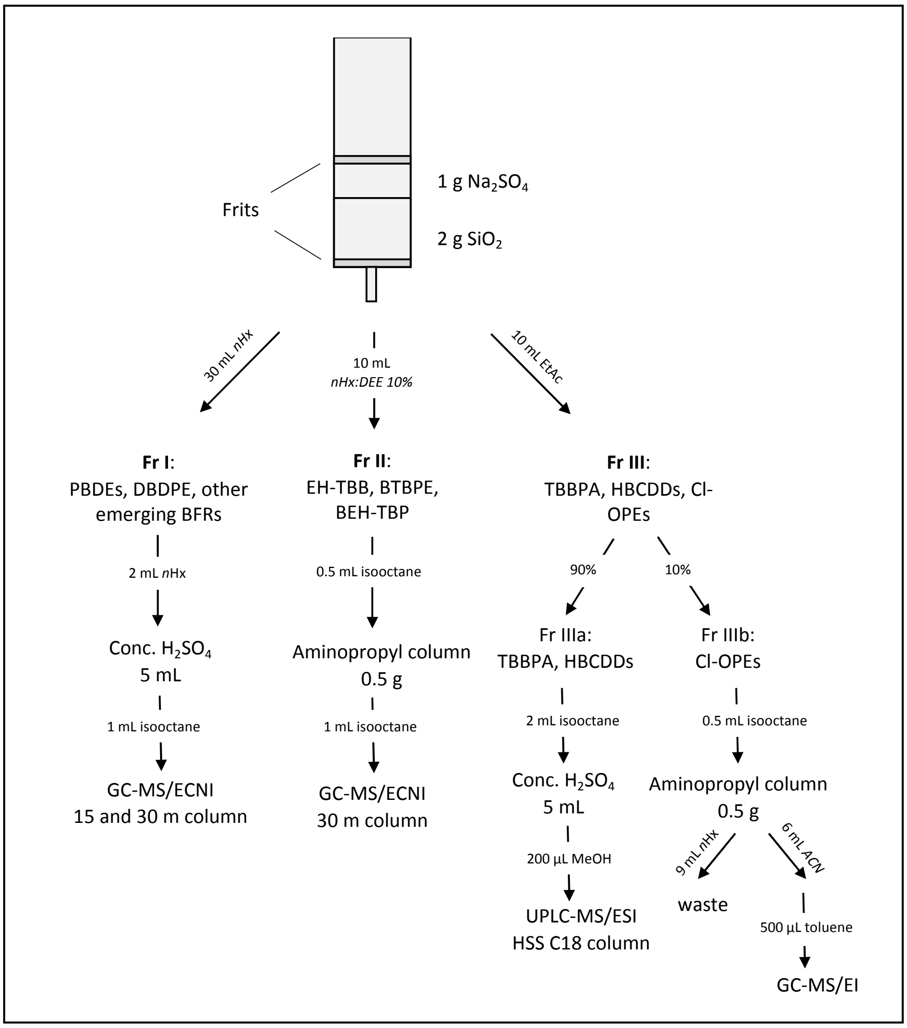

Figure S3. Clean-up method workflow for halogenated flame retardants (HFRs), adapted from Sahlström et al.[3]

Supplementary tables

Table S1. Target per- and polyfluoroalkyl substances (PFAS), chlorinated paraffins (CPs) and halogenated flame retardants (HFRs).

	Name	Acronym	Molecular formula	CASRN
PFAS extraction	Perfluorobutanoic acid	PFBA	$C_4F_7O_2H$	375-22-4
	Perfluoropentanoic acid	PFPeA	$C_5F_9O_2H$	2706-90-3
	Perfluorohexanoic acid	PFHxA	$C_6F_{11}O_2H$	307-24-4
	Perfluoroheptanoic acid	PFHpA	$C_7F_{13}O_2H$	375-85-9
	Perfluorooctanoic acid	PFOA^{L+Br}	$C_8F_{15}O_2H$	335-67-1
	Perfluorononanoic acid	PFNA	$C_9F_{17}O_2H$	375-95-1
	Perfluorodecanoic acid	PFDA	$C_{10}F_{19}O_2H$	335-76-2
	Perfluoroundecanoic acid	PFUnDA	$C_{11}F_{21}O_2H$	2058-94-8
	Perfluorododecanoic acid	PFDoDA	$C_{12}F_{23}O_2H$	307-55-1
	Perfluorotridecanoic acid	PFTrDA	$C_{13}F_{25}O_2H$	72629-94-8
	Perfluorotetradecanoic acid	PFTeDA	$C_{14}F_{27}O_2H$	376-06-7
	Perfluorobutane sulfonic acid	PFBS	$C_4F_9SO_3H$	375-73-5
	Perfluorohexane sulfonic acid	PFHxS^{L+Br}	$C_6F_{13}SO_3H$	355-46-4
	Perfluorooctane sulfonic acid	PFOS^{L+Br}	$C_8F_{17}SO_3H$	1763-23-1
	Perfluorodecane sulfonic acid	PFDS^{L+Br}	$C_{10}F_{21}SO_3H$	335-77-3
	Perfluorooctane sulfonamide	FOSA^{L+Br}	$C_8F_{17}SO_2NH_2$	754-91-6
CPs extraction	Very short chain chlorinated paraffins	vSCCPs	$C_{6\text{-}9}H_xCl_y$	na
	Short chain chlorinated paraffins	SCCPs	$C_{10\text{-}13}H_xCl_y$	na
	Medium chain chlorinated paraffins	MCCPs	$C_{14\text{-}17}H_xCl_y$	na
	Long chain chlorinated paraffins	LCCPs	$C_{18\text{-}30}H_xCl_y$	na
HFRs extraction	2,4,4'-Tribromodiphenyl ether	BDE28	$C_{12}H_7Br_3O$	41318-75-6
	3,3',4-Tribromodiphenyl ether	BDE35	$C_{12}H_7Br_3O$	147217-80-9
	2,2´,4,4´-Tetrabromodiphenyl ether	BDE47	$C_{12}H_6Br_4O$	5436-43-1
	2,2',4,5'-Tetrabromodiphenyl ether	BDE49	$C_{12}H_6Br_4O$	243982-82-3
	2,3',4,4'-Tetrabromodiphenyl ether	BDE66	$C_{12}H_6Br_4O$	189084-61-5
	2,2',3,4,4'-Pentabromodiphenyl ether	BDE85	$C_{12}H_5Br_5O$	182346-21-0
	2,2′,4,4′,5-Pentabromdiphenylether	BDE99	$C_{12}H_5Br_5O$	60348-60-9
	2,2′,4,4′,6-Pentabromdiphenylether	BDE100	$C_{12}H_5Br_5O$	189084-64-8
	2,2′,4,4′,5,5′-Hexabromdiphenylether	BDE153	$C_{12}H_4Br_6O$	68631-49-2
	2,2′,4,4′,5,6′-Hexabromdiphenylether	BDE154	$C_{12}H_4Br_6O$	207122-15-4
	2,2',3,4,4',5,6'-Heptabromodiphenyl ether	BDE182	$C_{12}H_3Br_7O$	442690-45-1
	2,2',3,4,4',5',6-heptabromodiphenyl ether	BDE183	$C_{12}H_3Br_7O$	207122-16-5
	2,2',3,3',4,4',5,6'-Octabromodiphenyl ether	BDE196	$C_{12}H_2Br_8O$	446255-39-6
	2,2',3,3',4,4',6,6'-octabromodiphenyl ether	BDE197	$C_{12}H_2Br_8O$	117964-21-3
	2,2',3,4,4',5,5',6-Octabromodiphenyl ether	BDE203	$C_{12}H_2Br_8O$	337513-72-1
	2,2',3,3',4,4',5,5',6-nonabromodiphenyl ether	BDE206	$C_{12}HBr_9O$	63387-28-0
	2,2',3,3',4,4',5,6,6'-nonabromodiphenyl ether	BDE207	$C_{12}HBr_9O$	437701-79-6
	2,2',3,3',4,5,5',6,6'-nonabromodiphenyl ether	BDE208	$C_{12}HBr_9O$	437701-78-5
	Decabromodiphenylether	BDE209	$C_{12}Br_{10}O$	1163-19-5
	Decabromodiphenyl ethane	DBDPE	$C_{14}H_4Br_{10}$	84852-53-9
	1,3,5-Tribromo-2-hydroxybenzene	TBP-AE	$C_9H_7Br_3O$	3278-89-5
	α-4-(1,2-Dibromoethyl)-1,2-dibromocyclohexane	α-DBE-DBCH	$C_8H_{12}Br_4$	3322-93-8
	β-4-(1,2-Dibromoethyl)-1,2-dibromocyclohexane	β-DBE-DBCH	$C_8H_{12}Br_4$	3322-93-8
	2-Bromoallyl 2,4,6-tribromophenyl ether	BATE	$C_9H_6Br_4O$	99717-56-3
	Pentabromobenzene	PBBz	C_6HBr_5	608-90-2
	Pentabromotoluene	PBT	$C_7H_3Br_5$	87-83-2
	1,2-Bis(2,4,6-tribromophenoxy)ethane	BTBPE	$C_{14}H_8Br_6O_2$	37853-59-1
	Hexabromobenzene	HBB	C_6Br_6	87-82-1
	Hexachlorocyclopentenyl-dibromocyclooctane	DBHCTD	$C_{13}H_{14}Br_2Cl_6$	51936-55-1
	syn-Dodecachlorodimethanodibenzocyclooctane	DDC-CO (syn)	$C_{18}H_{12}Cl_{12}$	13560-89-9
	anti-Dodecachlorodimethanodibenzocyclooctane	DDC-CO (anti)	$C_{18}H_{12}Cl_{12}$	13560-89-9
	2-Ethylhexyl-2,3,4,5-tetrabromobenzoate	EH-TBB	$C_{15}H_{18}Br_4O_2$	183658-27-7
	1,2-Bis(2,4,6-tribromophenoxy)ethane	BTBPE	$C_{14}H_8Br_6O_2$	37853-59-1
	Bis(2-ethylhexyl) tetrabromophthalate	BEH-TBP	$C_{24}H_{34}Br_4O_4$	26040-51-7
	Tetrabromobisphenol A	TBBPA	$C_{15}H_{12}Br_4O_2$	79-94-7
	α-Hexabromocyclododecane	α-HBCDD	$C_{12}H_{18}Br_6$	134237-50-6; 25637-99-4
	β-Hexabromocyclododecane	β-HBCDD	$C_{12}H_{18}Br_6$	678970-16-6; 25637-99-4
	γ-Hexabromocyclododecane	γ-HBCDD	$C_{12}H_{18}Br_6$	134237-52-8; 25637-99-4
	Tris(2-chloroethyl) phosphate	TCEP	$C_6H_{12}Cl_3O_4P$	115-96-8
	Tris(-1-chloro-2-propyl)phosphate	TCIPP	$C_9H_{18}Cl_3O_4P$	13674-84-5
	Tris(1,3-dichloropropan-2-yl) phosphate	TDCPP	$C_9H_{15}Cl_6O_4P$	13674-87-8

Table S2. Target PFAS with average retention times (RT), precursor, quantitative and qualitative ions, as well as internal standard (IS) used, and limits of quantification (LOQs).

	Acronym	RT (min)	Precursor ion	Quantitative product ion	Qualitative product ion	IS	LOQ (ng/g)
PFCAs	PFBA	0.78	213	169	149	$^{13}C_4$-PFBA	0.288
	PFPeA	1.67	263	219	169	$^{13}C_2$-PFHxA	0.082
	PFHxA	2.28	313	269	119	$^{13}C_2$-PFHxA	0.288
	PFHpA	2.70	363	319	169	$^{13}C_4$-PFOA	0.288
	$PFOA^{L+Br}$	3.04	413	369	169	$^{13}C_4$-PFOA	0.288
	PFNA	3.36	463	419	219	$^{13}C_5$-PFNA	0.288
	PFDA	3.66	513	469	269	$^{13}C_2$-PFDA	0.288
	PFUnDA	3.96	563	519	269	$^{13}C_2$-PFUnDA	0.288
	PFDoDA	4.25	613	569	169	$^{13}C_2$-PFDoDA	0.288
	PFTrDA	4.53	662.9	619	169	$^{13}C_2$-PFDoDA	0.288
	PFTeDA	4.8	712.9	669	169	$^{13}C_2$-PFDoDA	0.082
PFSAs	PFBS	2.25	298.9	80	99	$^{18}O_2$-PFHxS	0.254
	$PFHxS^{L+Br}$	3.11	398.9	80	99	$^{18}O_2$-PFHxS	0.272
	$PFOS^{L+Br}$	3.76	498.9	80	99	$^{13}C_4$-PFOS	0.078
	$PFDS^{L+Br}$	4.35	598.9	80	99	$^{13}C_4$-PFOS	0.278
	$FOSA^{L+Br}$	4.31	497.9	78	169	$^{13}C_8$-FOSA	0.292

$^{L+Br}$ = linear + branched. LOQS determined by the lowest calibration concentration that showed a well-shaped peak with intensity >1e3 and signal-to-noise >3.

Table S3. Spike/recovery experiment results for PFAS spiked at 5 ng in sludge (*n*=3).

Analyte	Spiking amount (ng)	Recoveries (%)		
		Average	Stdev	RSD
PFPeA	5	74.0	13.3	18%
PFHxA	5	80.2	14.4	18%
PFHpA	5	60.9	10.1	17%
L-PFOA	5	66.5	10.9	16%
PFNA	5	63.6	12.6	20%
PFDA	5	67.9	13.4	20%
PFUnDA	5	66.5	12.4	19%
PFDoDA	5	67.2	15.0	22%
**PFTrDA**	**5**	**249.2**	**46.5**	**19%**
**PFTeDA**	**5**	**323.8**	**44.7**	**14%**
PFBS	4.4	88.9	11.4	13%
PFHxS	4.7	71.2	12.4	17%
L-PFOS (80/99 combined)	4.8	76.7	25.2	33%
PFDS	4.8	72.6	4.9	7%
L-FOSA	5.1	70.5	15.7	22%

Severe over-recoveries are bolded. Note that these extracts were non-recovery corrected (Extract 2). This spiking experiment has previously been reported in Kärrman et al.[6]

Table S4. PFAS concentrations measured in NIST SRM 2781 domestic sludge, compared to NIST reported values and other studies. Note that PFAS results in this study were non-recovery corrected.

	This study (*n*=3) **non-recovery corrected**	NIST SRM 2781	Aro et al.[7] (*n*=3)	Aro et al.[8] (*n*=2)	Munoz et al.[9] (*n*=5)
	Average ± stdev (ng/g)	Non-certified values (ng/g)	Average ± CI (ng/g)	Average ± CI (ng/g)	(ng/g)
PFBA	nd				6.3 ± 0.3
PFPeA	6.3 ± 0.6				6.6 ± 0.4
PFHxA	9.5 ± 0.7	13	13.8 ± 1.4	14.4 ± 0.1	17 ± 0.9
PFHpA	5.9 ± 0.8	7.96	16.3 ± 1.0	22.2 ± 11.9	9.6 ± 0.5
L-PFOA	16 ± 0.8	28.5	31.8 ± 1.8	31.2 ± 2.4	25 ± 1.8
Br-PFOA	1.1 ± 0.1				
PFNA	4.8 ± 0.4				1.7 ± 0.3
PFDA	3.3 ± 0.2				
PFUnDA	2.0 ± 0.2				
PFDoDA	1.5 ± 0.1				
PFTrDA	0.4 ± 0.1				
PFTeDA	2.3 ± 0.2				
PFHxDA	21.6 ± 2.2				
PFOcDA	13.9 ± 2.8				
PFBS	nd				
PFHxS	8.6 ± 0.8	9.39	8.7 ± 0.0	8.4 ± 1.6	7.6 ± 0.3
L-PFOS	128.2 ± 3.7	225	206.6 ± 8.3	187.9 ± 21.8	240 ± 13
Br-PFOS	14.3 ± 1.2				
PFDS	97.8 ± 1.9				230 ± 11
FOSA	2.3 ± 2.4				7.4 ± 1.3
6:2 FTS	nd				2.0 ± 0.3
∑F_PFAS (ng F/g)	226.3 ± 10.0				
EOF (ng F/g dw)	3590 ± 311				

Table S5. Limits of quantification (LOQs) for CPs in sludge.

	LOQ (ng/g)[a]
∑CPs	87.85
vSCCPs	Not detected in blank
∑SCCPs	14.84
∑MCCPs	69.95
∑LCCPs	4.17

[a]LOQ calculated for the average amount of sludge used (0.5 g), using average concentration in the blank + 10 times the standard deviation.

Table S6. Spike/recovery experiment results for chlorinated paraffins in diatomaceous earth (*n*=3).

			% Recovery	
Instrument		Spiked amount (ng)	Average	Stdev
LC-HRMS	∑SCCPs 55.5 %Cl	196.1	89%	9%
	∑MCCPs 52.0 %Cl	1735.9	109%	10%
	∑LCCPs 49.0 %Cl	1068.0	102%	3%
CIC – extract	∑Cl_CP	1534.8	95%	

Table S7. NIST dust SRM 2585 comparison for chlorinated paraffins in µg/g.

	This study	Brits et al.[10]	Brandsma et al.[11]	Shang et al.[12]	Wong et al.[13]
∑vSCCPs	<LOD	na	na	na	na
∑SCCPs	6.49 ± 0.22	8.65 ± 0.16	7.1 ± 0.2	7.58 ± 0.43	102-119
∑MCCPs	11.0 ± 0.45	11.98 ± 0.24	10 ± 0.2	16.4 ± 2.1	116-121
∑LCCPs	19.6 ± 0.17	19.26 ± 0.56	16 ± 0.4	na	87-112
∑Cl_CPs ± stdev (µg Cl/ g dw)	17.9 ± 0.3				
EOCl_CPs ± stdev (µg Cl/g dw)	17.0 ± 1.6				

na = not analyzed

Table S8. Target HFRs with average retention times (RT), precursor and quantitative ions, internal standard (IS) used and limits of quantification (LOQs).

		Compound	RT (min)	Quantification ions (m/z)	IS	LOQs (ng/g)
Fr I -ECNI 30m	Tri-HeptaBDEs	BDE28	15.33	79, 81	^{13}C-bde155	0.020
		BDE35	15.70	79, 81	^{13}C-bde155	0.050
		BDE47	19.34	79, 81	^{13}C-bde155	0.049
		BDE49	18.66	79, 81	^{13}C-bde155	0.051
		BDE66	20.03	79, 81	^{13}C-bde155	0.049
		BDE85	26.90	79, 81	^{13}C-bde155	0.129
		BDE99	24.42	79, 81	^{13}C-bde155	0.050
		BDE100	22.91	79, 81	^{13}C-bde155	0.125
		BDE153	29.68	79, 81	^{13}C-bde155	0.166
		BDE154	28.05	79, 81	^{13}C-bde155	0.066
		BDE182	32.96	79, 81	^{13}C-bde155	0.127
		BDE183	32.95	79, 81	^{13}C-bde155	0.066
Fr I - ECNI 15m	OcNoBDEs	BDE196	11.08	407, 409	^{13}C-bde197	0.126
		BDE197	10.94	407, 409	^{13}C-bde197	0.124
		BDE203	11.04	79, 81	^{13}C-bde197	0.129
		BDE206	12.09	485, 487	^{13}C-bde197	0.259
		BDE207	11.89	485, 487	^{13}C-bde197	0.536
		BDE208	11.80	485, 487	^{13}C-bde197	0.207
	Deca - [illegible]	BDE209	13.49	484.6, 486.6	^{13}C-bde209	0.492
		DBDPE	14.69	79,81	^{13}C-bde209	1.232
Fr I - ECNI 30m	EFR I	TBP-AE	9.51	288.8, 290.8	^{13}C-bde155	0.432
		α-DBE-DBCH	11.99	79, 81	^{13}C-bde155	0.503
		β-DBE-DBCH	12.12	79, 81	^{13}C-bde155	0.503
		BATE	12.51	79, 81	^{13}C-bde155	0.494
		PBBz	12.96	392.6, 471.6	^{13}C-bde155	0.491
		PBT	15.42	79, 81	^{13}C-bde155	0.495
		BTBPE	17.87	250.8	^{13}C-bde155	2.461
		HBB	18.37	470.5, 472.5	^{13}C-bde155	0.523
		DBHCTD	24.95	79, 81	^{13}C-bde155	0.496
		DDC-CO (syn)	35.36	436, 438, 472, 474	^{13}C-bde155	0.502
		DDC-CO (anti)	36.28	436, 438, 472, 474	^{13}C-bde155	0.511
Fr II - ECNI [illegible]	EFR II	EH-TBB	24.61	79, 81, 469, 471	M-EH-TBB	0.499
		BTBPE	33.71	79, 81	M-BEHTBP	0.177
		BEH-TBP	34.78	79, 81, 513, 515	M-BEHTBP	1.166
Fr IIIa - LC		TBBPA	2.12	79, 81	M-TBBPA	0.100
		α-HBCDD	3.06	79, 81	^{13}C-α-HBCDD	0.050
		β-HBCDD	3.22	79, 81	^{13}C-β-HBCDD	0.051
		γ-HBCDD	3.32	79, 81	^{13}C-γ-HBCDD	0.049
Fr IIIb - EI	Cl-OPEs	TCEP	12.68	249, 251	dTCEP	2.430[a]
		TCIPP	13.32	277, 279	dTCEP	21.575[a]
		TDCPP	22.75	381, 383	dTDCPP	0.748

[a]Detected in procedural blanks.

LOQs were determined by the lowest calibration concentration that showed a well-shaped peak with intensity >1e3 and signal-to-noise (S/N) >3), unless when detected in blanks, then the LOQ was determined as the average of the quantified concentrations in the method blanks plus ten times the standard deviation.

Table S9. Internal standard (IS) yields for HFRs in blanks and sludge samples.

	Blanks *n*=5			Samples *n*=4		
IS	Average	Stdev	RSD	Average	Stdev	RSD
^{13}C-bde155	73%	12%	16%	64%	4%	7%
^{13}C-bde197	84%	14%	16%	64%	10%	16%
^{13}C-bde209	80%	13%	16%	49%	25%	52%
M-EH-TBB	95%	19%	20%	86%	10%	12%
M-BEHTBP	91%	24%	26%	68%	11%	16%
M-TBBPA	4%	1%	15%	27%	13%	47%
^{13}C-α-HBCDD	45%	14%	32%	233%	211%	91%
^{13}C-β-HBCDD	8%	2%	23%	47%	12%	26%
^{13}C-γ-HBCDD	8%	1%	18%	37%	16%	43%
dTCEP	13%	1%	7%	18%	3%	15%
dTDCPP	38%	4%	11%	46%	11%	23%

ISs marked in red were <20% or >120% and were marked as “not quantified”.

Table S10. Spike/recovery results for HFR analytes spiked in sludge (*n*=3). Recoveries <60% and <140% are bolded.

Analyte	Nominal spiking amount (ng)	Recoveries (%) n=3		
		Average	stdev	RSD
BDE28	50	nd		
BDE35	50	nd		
BDE47	50	102.1	9.1	9%
BDE49	50	111.7	9.5	9%
BDE66	50	108.8	11.7	11%
BDE85	50	104.4	14.9	14%
BDE99	50	133.4	19.8	15%
BDE100	50	125.5	13.5	11%
BDE153	50	127.6	23.9	19%
BDE154	50	127.9	19.2	15%
BDE182	50	91.6	20.7	23%
BDE183	50	99.8	19.1	19%
**BDE196**	**50**	**37.6**	**15.3**	**41%**
BDE197	50	78.6	2.6	3%
BDE203	50	74.6	13.3	18%
**BDE206**	**50**	**59.1**	**6.9**	**12%**
BDE207	50	67.9	4	6%
BDE208	50	73.1	4.9	7%
BDE209	1000	88.4	1.7	2%
**DBDPE**	**1000**	**177.7**	**21.4**	**12%**
**TBP-AE**	**50**	**6.3**	**4.2**	**67%**
α-DBE-DBCH	50	102	7.6	7%
β-DBE-DBCH	50	101.8	7.6	7%
BATE	50	87.9	4.8	5%
**PBBz**	**50**	**198.5**	**21.4**	**11%**
PBT	50	75.3	8.8	12%
**TBP-DBPE**	**50**	**261**	**8.8**	**3%**
**HBB**	**50**	**250.5**	**23.1**	**9%**
**DBHCTD**	**50**	**407.3**	**44.9**	**11%**
**DDC-CO (syn)**	**50**	**160.6**	**24.1**	**15%**
DDC-CO (anti)	50	98.9	25.6	26%
EH-TBB	50	108.4	6.9	6%
**BTBPE**	**50**	**42.3**	**2.7**	**6%**
**BEH-TBP**	**1000**	**21.7**	**4**	**18%**

Table S11. Eluent gradient for EOX analysis.

Time	OH^- concentration (mM)
0	8
4	8
14	45
14.1	60
16	60
16.1	8
31	8

Flow rate 0.25 ml/min.

Table S12. Results NaF spike/recovery experiment.

n	ID	**Recovery (%)**		**Sludge concentration (µg F/g)**	
		**Average**	**stdev**	**Average**	**stdev**
3	[a]Blank	-	-	0.010	0.002
3	[a]Sludge no spike	-	-	0.017	0.006
3	[a]Sludge 0.25 mg spike F-	1.25%	0.16%	5.98	0.54
3	[a]Sludge + 23 mg spike F- (50 mg NaF)	0.04%	0.06%	17.50	24.90
2	Reference standard 0.25 mg spike F-<br>(no extraction; spike + 600 µL methanol + 6 mL water)	157%	0%	-	-
2	Reference standard 23 mg spike F-<br>(no extraction; spike + 600 µL methanol + 6 mL water)	121%	32%	-	-
3	[b]Water extraction<br>(sludge + water + ultrasonic bath + centrifuge)	-	-	4.01	0.27

[a]Samples were extracted in the same manner as the PFAS extraction, only the final extract (600 µL methanol) was diluted with 6 mL deionized water to enable F- measurements with ISE (ISEF121; 05/2021, Edition 5; Hach Company).
[b]Sludge samples extracted with water only.

Table S13. Combustion efficiencies measured with CIC for organic standard solutions.

Compound	Nominal halogen equivalent concentration (ng X/µL)	Recovery (%)	Average ± stdev (%)
PFOS (ng F/µL)	0.25	92%	85 ± 6%
	0.5	85%	
	2	84%	
	5	76%	
	10	89%	
PFOA (ng F/µL)	0.25	97%	91 ± 4%
	0.5	89%	
	2	86%	
	5	91%	
	10	94%	
TCIPP (ng Cl/µL)	0.15	<DL	84 ± 16%
	0.35	108%	
	1	82%	
	3	73%	
	8.5	74%	
TDCPP (ng Cl/µL)	0.15	<DL	96 ± 25%
	0.5	77%	
	1.2	132%	
	3.5	85%	
	10	90%	
SCCPs (55.5% from Dr Ehernstorfer GmbH)	55.5	85 ± 4% (*n=3*)	91 ± 6%
MCCPs (52.0% from Dr Ehernstorfer GmbH)	52.0	99 ± 3% (*n=3*)	
LCCPs (49.0% from Dr Ehernstorfer GmbH)	49.0	90 ± 1% (*n=3*)	
∑CP-mix	51.16	92 ± 2% (*n=3*)	
BDE209 (ng Br/µL)	0.05	<DL	110 ± 31%
	0.15	<DL	
	0.5	74%	
	1.5	127%	
	4.2	129%	
BDE183 (ng Br/µL)	0.4	<DL	137 ± 40%
	0.75	93%	
	2	150%	
	7	169%	

Table S14. Contribution of chlorinated paraffin homologue profiles (in %) in sewage sludge.

	Cl3	Cl4	Cl5	Cl6	Cl7	Cl8	Cl9	Cl10	Cl11	Cl12	Cl13
C6											
C7											
C8		<0.00									
C9			0.01	0.01	<0.00						
C10			0.08	0.26	0.14	0.03	<0.00				
C11	<0.00	<0.00	0.22	0.62	0.55	0.18	0.04	<0.00			
C12	<0.00	<0.00	0.26	0.70	0.74	0.37	0.11	0.01	<0.00		
C13	<0.00	0.01	0.46	1.28	1.42	0.68	0.17	0.05	<0.00		
C14	<0.00	0.21	3.96	10.06	10.72	5.24	1.32	0.22	0.01		
C15		0.17	2.44	6.40	7.26	4.26	1.26	0.21	0.02	<0.00	
C16	<0.00	0.10	1.36	3.98	4.14	3.12	1.07	0.24	0.03	<0.00	
C17		0.08	1.02	2.20	2.90	1.94	1.18	0.31	0.04	<0.00	
C18		0.09	0.64	1.44	1.44	1.18	0.70	0.20	0.05	<0.00	
C19		0.04	0.19	0.35	0.45	0.34	0.31	0.08	0.02	<0.00	
C20		0.04	0.24	0.35	0.38	0.23	0.17	0.08	0.05	<0.00	
C21		0.02	0.11	0.12	0.13	0.12	0.06	0.04	0.01	<0.00	
C22		0.02	0.15	0.28	0.17	0.19	0.11	0.09	0.02	0.01	
C23		0.01	0.10	0.12	0.15	0.11	0.07	0.03	0.01	<0.00	
C24		0.01	0.12	0.20	0.15	0.20	0.11	0.06	0.02	0.01	<0.00
C25			0.05	0.08	0.12	0.08	0.06	0.03	0.01	<0.00	
C26		<0.00	<0.00	0.05	0.07	0.07	0.10	0.06	0.03	0.01	<0.00
C27			0.03	0.04	0.06	0.05	0.04	0.02	<0.00		
C28			0.03	0.06	0.05	0.04	0.03	0.02	<0.00	<0.00	<0.00
C29			0.02	0.04	0.02	0.02	0.02	0.01	<0.00		
C30			<0.00	0.03	0.02	0.02	0.01	0.01	<0.00		
C31				0.01	0.01	0.01	0.01	0.01	<0.00		
C32				<0.00	0.01	<0.00	<0.00	<0.00	<0.00		
C33											

Table S15. Concentrations of OPEs in WWTP sludge (ng/g dw) from literature.

			Conc. ng/g dw		
	Sampling country	Sampling year	TCEP	TCPP	TDCPP
Marklund et al.[14]	Sweden	2002-2003	6.6-110	61-1900	3.0-260
Haglund et al.[15]	Sweden	2012	7-34	1800-4000	290-970
	Sweden	2013	9.2-26	2500-6400	250-830
Haglund et al.[16]	Sweden	2016	4.5-15	650-4600	90-250
	Sweden	2017	7.1-44	520-4100	28-240
**This study**	**Sweden**	**2019**	**nq**	**60.3±8.7**	**4.2±0.5**
Wang et al.[17]	US	2006-2007	10.6	61.7	101
Zeng et al.[18]	China	2011	646.6	562.7	64

Table S16. Mass balance overview table.

		TARGET ANALYSIS		MASS BALANCE			
	Target compound	Sludge concentration ± stdev (ng/g dw)		Sludge concentration ± stdev (ng/g dw)	∑F_PFAS ± stdev (ng F/g dw)	EOF ± stdev (ng F/g dw)	% unknown EOF average (range)
		Recovery corrected		Non-recovery corrected			
PFAS extraction n=3	PFBA	NF		<0.288			
	PFPeA	NF		NF			
	PFHxA	NF		<0.288			
	PFHpA	NF		<0.288			
	PFOA	<0.288		<0.288			
	PFNA	<0.288		<0.288			
	PFDA	0.97 ± 0.02		0.69 ± 0.10			
	PFUnDA	0.52 ± 0.10		0.45 ± 0.08			
	PFDoDA	0.71 ± 0.10		0.55 ± 0.06	6.40 ± 1.04	304 ± 116	98%
	PFTrDA	0.58 ± 0.18		0.36 ± 0.05			
	PFTeDA	0.84 ± 0.21		0.44 ± 0.12			
	PFBS	NF		<0.254			
	PFHxS	NF		0.62 ± 0.26			
	PFOS (linear + branched)	7.23 ± 0.55		5.12 ± 1.04			
	PFDS	NF		<0.278			
	FOSA	NF		<0.292			
					∑Cl_CPs ± stdev (ng Cl/ g dw)	EOCl_CPs ± stdev (ng Cl/g dw)	% unknown EOCl average (range)
CPs extraction (n=5)	∑vSCCP (C6-9)	-		5.51 ± 0.54			
	∑SCCP (C10-13)	-		181 ± 20.6	951 ± 169	1030 ± 417	8% (0-46%)
	∑MCCP (C14-17)	-		1127 ± 203			
	∑LCCP (C18-33)	-		502 ± 114			
					∑Cl_FRs ± stdev (ng Cl/ g dw)	EOCl_FRs ± stdev (ng Cl/g dw)	% unknown EOCl average (range)
HFR extraction ; Fr IIIb	TCEP	nq		nq			
	TCIPP	60.3 ± 8.7		<21.6	<7.4	633.9 ± 207.6	100%
	TDCPP	4.2 ± 0.5		<0.75			
					∑Br_BFRs ± stdev (ng Br/g dw)	EOBr ± stdev (ng Br/g)	% unknown EOBr average (range)
HFR extraction; Fr I -ECNI 30m	BDE28	0.07 ± 0.01		<0.020			
	BDE35	<0.050		<0.050			
	BDE47	3.53 ± 0.3		2.49 ± 0.43			
	BDE49	0.34 ± 0.02		0.14 ± 0.12			
	BDE66	<0.098		<0.098			
	BDE85	<0.129		<0.129			
	BDE99	4.53 ± 0.34		3.63 ± 0.65			
	BDE100	0.82 ± 0.02		0.64 ± 0.11			
	BDE153	0.43 ± 0.06		0.34 ± 0.07			
	BDE154	0.35 ± 0.01		0.3 ± 0.05			
	BDE182	NF		NF			
	BDE183	NF		NF			
HFR extraction; Fr I - ECNI 15m	BDE196	<0.251		<0.251			
	BDE197	0.16 ± 0.05		<0.251			
	BDE203	0.15 ± 0.07		0.15 ± 0.04			
	BDE206	1.51 ± 0.34		1.41 ± 0.14	93.9 ± 23.0	149 ± 10	37% (16-55%)
	BDE207	1.92 ± 0.67		0.94 ± 0.36			
	BDE208	1.23 ± 0.45		0.62 ± 0.16			
	BDE209	129.9 ± 10.4		80.5 ± 20.6			
	DBDPE	60.2 ± 7.3		23.2 ± 5.26			
HFR extraction; Fr I - ECNI 30m	TBP-AE	NF		NF			
	α-DBE-DBCH	NF		NF			
	β-DBE-DBCH	NF		NF			
	BATE	NF		NF			
	PBBz	NF		NF			
	PBT	<0.989		<0.989			
	BTBPE	NF		NF			
	HBB	NF		NF			
	DBHCTD	NF		NF			
	DDC-CO (syn)	NF		NF			

	DDC-CO (anti)	NF		NF			
HFR extraction ; Fr II - ECNI	EH-TBB	4.9 ± 1.19		4.6 ± 1.7	7.37 ± 1.94	38.9 ± 4.0	81% (73-87%)
	BTBPE	3.02 ± 0.77		1.0 ± 0.2			
	BEH-TBP	30.02 ± 7.32		8.9 ± 1.9			
HFR extraction; Fr IIa - LC	TBBPA	nq		<1.003	<1.71	<53.1	-
	α-HBCDD	nq		<0.495			
	β-HBCDD	nq		<0.507			
	γ-HBCDD	nq		<0.493			

NF = Not found, nq = not quantified, due to low IS recovery

References

(1) Yuan, B.; Wang, Y. W.; Fu, J. J.; Zhang, Q. H.; Jiang, G. Bin. An Analytical Method for Chlorinated Paraffins and Their Determination in Soil Samples. *Chinese Sci. Bull.* **2010**, *55* (22), 2396–2402. https://doi.org/10.1007/s11434-010-3261-x.

(2) Nylund, K.; Asplund, L.; Jansson, B.; Jonsson, P.; Litzen, K.; Sellström, U. Analysis of Some Polyhalogenated Organic Pollutants in Sediment and Sewage Sludg. *Chemosphere* **1992**, *19* (12), 463–466. https://doi.org/10.20595/jjbf.19.0_3.

(3) Sahlström, L.; Sellström, U.; De Wit, C. A. Clean-up Method for Determination of Established and Emerging Brominated Flame Retardants in Dust. *Anal. Bioanal. Chem.* **2012**, *404* (2), 459–466. https://doi.org/10.1007/s00216-012-6160-y.

(4) Hites, R. A. Polybrominated Diphenyl Ethers in the Environment and in People: A Meta-Analysis of Concentrations. *Environ. Sci. Technol.* **2004**, *38* (4), 945–956. https://doi.org/10.1021/es035082g.

(5) Stockholm Vatten. Henriksdals avloppsreningsverk: För stockholmarnas och miljöns bästa https://www.stockholmvattenochavfall.se/globalassets/pdf1/avloppsvatten/henriksdals-reningsverk/henriksdals-reningsverk (accessed Aug 17, 2021).

(6) Kärrman, A.; Yeung, L. W. Y.; Spaan, K. M.; Lange, F. T.; Nguyen, M. A.; Plassmann, M.; de Wit, C. A.; Scheurer, M.; Awad, R.; Benskin, J. P. Can Determination of Extractable Organofluorine (EOF) Be Standardized? First Interlaboratory Comparisons of EOF and Fluorine Mass Balance in Sludge and Water Matrices. *Environ. Sci. Process. Impacts* **2021**. https://doi.org/10.1039/d1em00224d.

(7) Aro, R.; Eriksson, U.; Kärrman, A.; Chen, F.; Wang, T.; Yeung, L. W. Y. Fluorine Mass Balance Analysis of Effluent and Sludge from Nordic Countries. *ACS ES&T Water* **2021**, *1* (9), 2087–2096. https://doi.org/10.1021/acsestwater.1c00168.

(8) Aro, R.; Carlsson, P.; Vogelsang, C.; Kärrman, A.; Yeung, L. W. Fluorine Mass Balance Analysis of Selected Environmental Samples from Norway. *Chemosphere* **2021**, *283*. https://doi.org/10.1016/j.chemosphere.2021.131200.

(9) Munoz, G.; Michaud, A. M.; Liu, M.; Vo Duy, S.; Montenach, D.; Resseguier, C.; Watteau, F.; Sappin-Didier, V.; Feder, F.; Morvan, T.; Houot, S.; Desrosiers, M.; Liu, J.; Sauvé, S. Target and Nontarget Screening of PFAS in Biosolids, Composts, and Other Organic Waste Products for Land Application in France. *Environ. Sci. Technol.* **2021**. https://doi.org/10.1021/acs.est.1c03697.

(10) Brits, M.; de Boer, J.; Rohwer, E. R.; De Vos, J.; Weiss, J. M.; Brandsma, S. H. Short-, Medium-, and Long-Chain Chlorinated Paraffins in South African Indoor Dust and Cat Hair. *Chemosphere* **2020**, *238*, 124643. https://doi.org/10.1016/j.chemosphere.2019.124643.

(11) Brandsma, S. H.; Brits, M.; Groenewoud, Q. R.; Van Velzen, M. J. M.; Leonards, P. E. G.; De Boer, J. Chlorinated Paraffins in Car Tires Recycled to Rubber Granulates and Playground Tiles. *Environ. Sci. Technol.* **2019**, *53* (13), 7595–7603. https://doi.org/10.1021/acs.est.9b01835.

(12) Shang, H.; Fan, X.; Kubwabo, C.; Rasmussen, P. E. Short-Chain and Medium-Chain Chlorinated Paraffins in Canadian House Dust and NIST SRM 2585. *Environ. Sci. Pollut. Res.* **2019**, *26* (8), 7453–7462. https://doi.org/10.1007/s11356-018-04073-2.

(13) Wong, F.; Suzuki, G.; Michinaka, C.; Yuan, B.; Takigami, H.; de Wit, C. A. Dioxin-like Activities, Halogenated Flame Retardants, Organophosphate Esters and Chlorinated Paraffins in Dust from Australia, the United Kingdom, Canada, Sweden and China. *Chemosphere* **2017**, *168*, 1248–1256. https://doi.org/10.1016/j.chemosphere.2016.10.074.

(14) Marklund, A.; Andersson, B.; Haglund, P. Organophosphorus Flame Retardants and Plasticizers in Swedish Sewage Treatment Plants. *Environ. Sci. Technol.* **2005**, *39* (19), 7423–7429. https://doi.org/10.1021/es051013l.

(15) Haglund, P. *Miljöövervakning Av Utgående Vatten & Slam Från Svenska Avloppsreningsverk*; 2015.

(16) Haglund, P. Miljöövervakning Av Utgående Vatten & Slam Från Svenska Avloppsreningsverk. **2019**.

(17) Wang, Y.; Kannan, P.; Halden, R. U.; Kannan, K. A Nationwide Survey of 31 Organophosphate Esters in Sewage Sludge from the United States. *Sci. Total Environ.* **2019**, *655*, 446–453. https://doi.org/10.1016/j.scitotenv.2018.11.224.

(18) Zeng, X.; Liu, Z.; He, L.; Cao, S.; Song, H.; Yu, Z.; Sheng, G.; Fu, J. The Occurrence and Removal of Organophosphate Ester Flame Retardants/Plasticizers in a Municipal Wastewater Treatment Plant in the Pearl River Delta, China. *J. Environ. Sci. Heal. - Part A Toxic/Hazardous Subst. Environ. Eng.* **2015**, *50* (12), 1291–1297. https://doi.org/10.1080/10934529.2015.1055158.
